# Efficacy of repetitive transcranial magnetic stimulation for insomnia disorder: a systematic review and meta-analysis of randomized controlled trials

**DOI:** 10.3389/fnins.2026.1816963

**Published:** 2026-06-01

**Authors:** ZhiGang Cao, Qun Shi, ZhengRong Shi, DanYang Yuan, Sha Zeng

**Affiliations:** 1 Department of Neurology, Sichuan Taikang Hospital, Chengdu, Sichuan, China; 2 Department of Rehabilitation Medicine, Xianyang Hospital of Yan'an University, Xianyang, China; 3 Department of Traditional Chinese Medicine Pharmacy, Chengdu Integrated TCM and Western Medicine Hospital, Chengdu, Sichuan, China; 4 Department of Rehabilitation Medicine, Sichuan Taikang Hospital, Chengdu, Sichuan, China

**Keywords:** efficacy, insomnia disorder, meta-analysis, randomized controlled trial, repetitive transcranial magnetic stimulation

## Abstract

**Objective:**

Insomnia Disorder (ID) is associated with significant health burdens. First-line treatments are limited by accessibility or side effects, necessitating alternative approaches. rTMS, a noninvasive neuromodulation technique, has shown promise in treating various neuropsychiatric disorders by modulating cortical excitability. This comprehensive meta-analysis explores the effect of rTMS on ID and identifies possible factors that influence it.

**Methods:**

A comprehensive search of the Cochrane Library, Embase, Web of Science, PubMed, CNKI, and Wanfang databases identified RCTs evaluating the effects of rTMS on insomnia disorder. Data synthesis and subgroup analysis were performed via SMD, WMD, relative risk (RR), and 95% CI to evaluate the effects of rTMS and its influencing factors. The review protocol was prospectively registered in PROSPERO (CRD42024626833).

**Results:**

Nineteen studies contributed 23 trials involving 1,690 adult participants. The rTMS group demonstrated markedly improved sleep quality compared with sham rTMS recipients in individuals with insomnia disorder. (PSQI total scores; ISI; *p* < 0.001); (PSG (SE); *p* = 0.003). Combined rTMS and medication were more effective than medication alone. (PSQI total scores; *p* = 0.003). In the subgroup analysis, after excluding a study with high heterogeneity, the rTMS cohort showed greater improvement in sleep quality than the other treatment groups. (PSQI total scores; *p* = 0.03).

**Conclusion:**

Independent rTMS and rTMS-medication combinations significantly improve sleep patterns and rest quality in patients with Insomnia Disorder. The safety and efficacy of LF-rTMS are also significant. The duration of the disease, treatment duration, and stimulation site may influence the sleep quality of patients with ID.

## Introduction

Insomnia disorder (ID) is characterized by difficulty initiating or maintaining sleep, waking too early, or poor sleep quality, leaving individuals unrefreshed (Seow et al., 2018). An estimated 16.2% of the global adult population, roughly 852 million people, meets the diagnostic criteria for insomnia disorder, with about 7.9%, or roughly 415 million, affected by severe cases of insomnia (Benjafield et al., 2025). The impact of insomnia disorder extends beyond just broken sleep, notably increasing the risk of mental health issues, interfering with daytime function, and adding to overall health concerns. Specifically, sleep problems are a main symptom in about 68% of people with anxiety disorders and 45% of those with Alzheimer’s disease (Hertenstein et al., 2019; Irwin and Vitiello, 2019). Patients often report fatigue and drowsiness, difficulty concentrating, and memory impairment, all of which reduce daily productivity (Ustinov et al., 2010). Additionally, Insomnia also compromises cardiovascular and metabolic wellness, along with immune system problems (Tobaldini et al., 2017). Although not classified as a critical illness, insomnia imposes a significant socioeconomic burden due to its indirect effects and the substantial costs it places on healthcare systems. A recent study found that insomnia costs more than $100 billion annually, driven primarily by indirect costs such as reduced workplace productivity, higher healthcare costs, and increased accident risk (Wickwire et al., 2016). Thus, insomnia disorder is a serious mental disorder that requires appropriate treatment. Currently, there are many methods for treating insomnia, including pharmaceutical (Wichniak et al., 2017) and non-medication approaches. While medications like benzodiazepines, non-benzodiazepine sleep aids, melatonin receptor agonists, and sedating antidepressants are often recommended to treat insomnia, their long-term use is limited by adverse side effects, addiction risks, and complex withdrawal processes (Wichniak et al., 2017; Atkin et al., 2018; Poza et al., 2022; Dekosky and Williamson, 2020; Khong et al., 2004). The range of non-pharmacological treatments includes CBT, phototherapy, acupuncture, TCM (traditional Chinese medicine), and non-invasive neurostimulation (Kim et al., 2021; Hertenstein et al., 2022; Chambe et al., 2023; Krone et al., 2025). Meanwhile, numerous research studies have demonstrated that Cognitive Behavioral Therapy for Insomnia (CBT-I) interventions have produced significant results in managing insomnia across diverse population groups (Chan et al., 2021). However, the dissemination of CBT-I is limited by a shortage of practitioners and financial barriers, which reduce its accessibility and acceptance among most patients with chronic insomnia, hindering its widespread adoption (Koffel et al., 2018). Furthermore, acupuncture and moxibustion, along with TCM, exhibit inherent limitations, as their effectiveness can vary depending on the practitioner’s skill (Xu et al., 2023). The limitations of current pharmacological and cognitive-behavioral treatments for insomnia have led to the search for safe, effective, and short-term non-drug alternatives. In this context, rTMS, an external brain-stimulation technique, has attracted increasing attention as a promising treatment.

rTMS uses noninvasive magnetic stimulation to induce electrical currents in the targeted cortical area. This process modulates the excitability of the stimulation site and nearby brain regions, thereby affecting regional brain blood flow and neurotransmitter activity (Klomjai et al., 2015). The after-effects of rTMS depend on the stimulation frequency and duration. Low-frequency stimulation (<1 Hz) exerts inhibitory effects, whereas high-frequency stimulation (>5 Hz) elicits excitatory effects in the brain (Pallanti, 2021). rTMS has been reported to have therapeutic benefits in various neurological and psychiatric conditions, including cerebrovascular accidents, Parkinson’s, movement disorders, depressive disorders, and psychotic conditions (Mcclintock et al., 2018; Safdar et al., 2023; Deng et al., 2025). Insomnia results from a complex interaction among psychological hyperarousal, circadian rhythm changes, and homeostatic processes, involving coordinated activity across multiple brain centers that regulate sleep–wake cycles. The ARAS (ascending reticular activating system) enhances alertness, and the VLPO (ventrolateral preoptic nucleus) facilitates drowsiness (Fuller et al., 2006). During wakefulness, the ARAS suppresses VLPO activity through cholinergic LH (lateral hypothalamus) neurons, monoaminergic pathways, and orexin-producing nuclei. Conversely, during sleep, the VLPO suppresses the ARAS via two principal inhibitory neuromodulators: gamma-aminobutyric acid (GABA) and galanin (Saper et al., 2010). GABA primarily promotes sleep, whereas norepinephrine and dopamine enhance alertness. Serotonin plays a dual role, being essential for both healthy sleep and wakefulness (Lu et al., 2006). Among pathological models of sleep disturbances characterized by hyperarousal, it is widely recognized as a common pathophysiological mechanism (Riemann et al., 2010). Studies using rTMS to examine insomnia have shown abnormal cortical excitability in individuals with insomnia (Lanza et al., 2015). Reducing hyperarousal in patients with insomnia may effectively improve their sleep (Van Der Werf et al., 2010). rTMS shows significant effectiveness in treating various medical conditions by modulating cortical activity and altering neurotransmitter levels in the brain. Research shows that both brain-derived neurotrophic factor (BDNF) and gamma-aminobutyric acid (GABA) are essential for sleep regulation. BDNF is particularly important because it improves the performance of GABAergic neurons (Mcallister, 2001). Research suggests that rTMS may enhance sleep patterns in individuals with ID by modulating neurotransmitter systems such as GABA, serotonin, and BDNF, as well as by increasing blood flow to the brain, thereby helping to maintain the body’s natural sleep cycle rhythms (Feng et al., 2019). Exposure to magnetic fields can affect the production and release of melatonin in the pineal gland, thereby influencing the levels of neurotransmitters such as serotonin (5-HT), norepinephrine (NE), and acetylcholine (ACh). These elements are essential for maintaining regular sleep patterns and physiological equilibrium (Jiang et al., 2013). High-frequency repetitive transcranial magnetic stimulation (HF-rTMS) can increase cortical excitability. Conversely, low-frequency repetitive transcranial magnetic stimulation (LF-rTMS) may decrease neuronal activity and promote both long-term potentiation (LTP) and long-term depression (LTD). As a result, this technique demonstrates efficacy in maintaining a lasting effect on cortical excitability and neural connectivity (Klomjai et al., 2015). Recent studies indicate that LF-rTMS applied to the sensory cortex can reduce neural activity, alleviate excessive alertness in patients, and elicit slow-wave patterns similar to those observed during normal sleep. Additionally, it improves the function of GABAergic neural networks and extends sleep duration (Joseph et al., 2024). rTMS, a non-invasive neurostimulation method, demonstrates therapeutic promise for ID. Especially when traditional therapies face limitations, its targeted mechanism provides a new alternative for addressing this growing clinical challenge.

Extensive research demonstrates that rTMS improves sleep quality in people with sleep disorders by modifying sleep patterns, enhancing daytime functioning and mood, and affecting activity in brain regions associated with sleep and their neurotransmitter systems (Van Der Werf et al., 2010; Mcallister, 2001; Shi and Hui, 2025). Although growing evidence supports the therapeutic potential of rTMS for sleep disorders, current research is limited in several respects, raising concerns about its clinical effectiveness and practical relevance. Major issues include transient therapeutic effects, variable responses among individuals, inconsistent stimulation parameters, and the absence of personalized protocols tailored to specific causes or stages of disease progression. These uncertainties mainly stem from methodological limitations in randomized controlled trials, such as small sample sizes, incomplete outcome assessments, and the absence of neuroimaging or biomarker-based measures (Joseph et al., 2024; Rosenquist and Mccall, 2019). Future clinical trials should focus on several key research directions. First, explore personalized targets such as default mode network nodes, the insula, and the anterior cingulate cortex, based on functional connectivity analyses from fMRI (Functional magnetic resonance imaging), EEG (electroencephalography), or fNIRS (functional near-infrared spectroscopy), and systematically compare their effectiveness (Cao et al., 2021). Second, develop standardized rTMS protocol parameters tailored to specific sleep disorder subtypes and thoroughly evaluate the effects of different frequencies, modalities, and treatment durations (Krone et al., 2025). Third, identify biomarkers to personalize treatment by integrating multidimensional data—including behavioral, neuroimaging, EEG, and genetic measures—to improve understanding of how rTMS works through multimodal indicators (Guo et al., 2023). Lastly, examine the combined effects of rTMS with CBT-I or pharmacotherapy, and evaluate safety and effectiveness.

Although current research on rTMS for ID has shown promising results, several limitations, such as inconsistent inclusion criteria and methodologies, create uncertainty about its therapeutic effectiveness and safety. A major issue is the high variability among study populations, which often include various diagnostic groups. For example, Nardone et al. (2020) recruited individuals with RLS (restless legs syndrome) and OSAS (obstructive sleep apnea syndrome), and depressed adolescents in their rTMS trial. These broad inclusion criteria introduce confounding variability, which reduces the clinical specificity of the reported treatment effects. Furthermore, the effectiveness of rTMS depends primarily on protocol parameters, including stimulation frequency, target site, and treatment duration. Systematic research into how these variables influence outcomes is essential for optimizing rTMS protocols and developing personalized treatment strategies. rTMS versus sham treatment for primary insomnia was effective in improving PSQI (Pittsburgh Sleep Quality Index) scores (Jiang et al., 2019). However, the objective measurement of ID by rTMS has not yet been fully summarized. Additionally, factors influencing rTMS therapeutic efficacy for insomnia remain underexplored.

Based on the background outlined above, this meta-analysis systematically assesses the therapeutic effectiveness of rTMS in ID through a rigorous review of RCTs. Specifically examines the impact of rTMS on neuroplasticity and functional recovery in patients with ID, aiming to carefully assess its safety and effectiveness for primary insomnia, whether used alone or combined with other treatments. Our research delivers robust data to guide clinical practice and refine treatment approaches. Moreover, subgroup analyses will examine potential moderators of treatment effects.

## Methods

**Figure 1:**
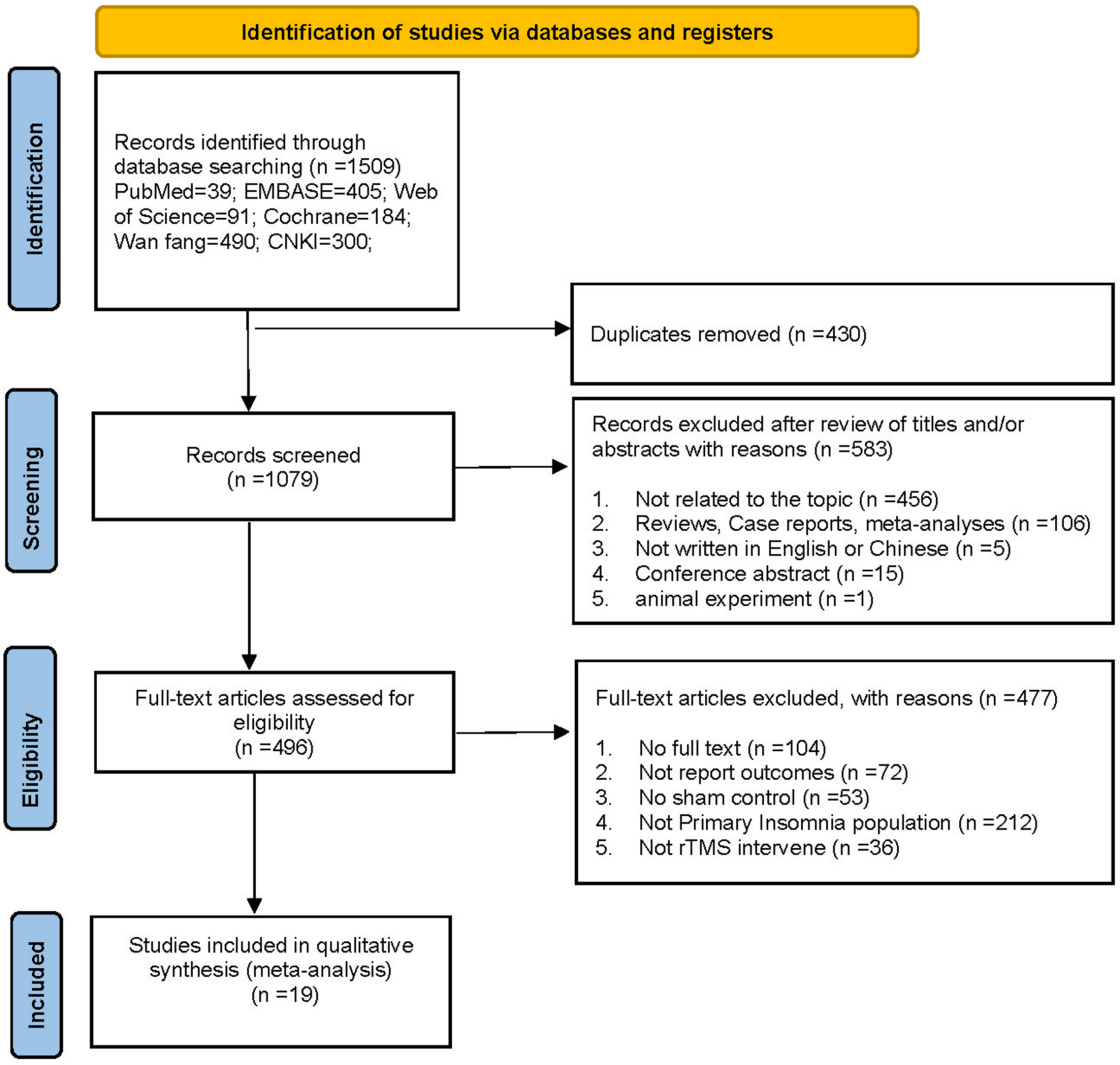
PRISMA. Flow diagram showing the search and selection procedure that was used for this meta-analysis. Diagram adapted from Page et al. (2021). rTMS, repetition transcranial magnetic stimulation.

Our meta-analysis is formally documented on PROSPERO under reference number (CRD42024626833), and it was developed in accordance with the Preferred Reporting Items for Systematic Reviews and Meta-Analyses (PRISMA) statement (Page et al., 2021; Figure 1) and the guidelines detailed in the Cochrane Handbook for Systematic Reviews of Interventions.

### Search strategy

Our search strategy combined thematic keywords and free-text terms, with adjustments informed by the search results. Our search involved a comprehensive review of six major databases—Embase, Web of Science, the Cochrane Library, PubMed, the China National Knowledge Infrastructure (CNKI), and the Wanfang Database—to collect clinical RCTs on rTMS for Insomnia Disorder, covering entries from the earliest records up to 13 October 2025. The search criteria included Mesh terms, free-text keywords, and their equivalents, such as transcranial magnetic stimulation, Sleep Onset and Maintenance Disorders, Early Morning Rises, Insomnia, Sleep Onset Insomnia, Persistent Insomnia, Sleep Disruptions, and randomized controlled trial. The specific search formula is provided in Supplementary material 1, which outlines the search terms and the search strategies. EndNote X9 is used to manage the retrieved literature.

### Literature inclusion criteria

The PICO (Da Costa Santos et al., 2007) framework specifies the essential components of this systematic review: P (Patients), I (Intervention), C (Comparison), and O (Outcomes).

P: Individuals 18 years and older diagnosed with primary sleeplessness according to established diagnostic frameworks—including the International Classification of Diseases, Tenth Edition (ICD-10) (Rust, 2010), the Diagnostic and Statistical Manual of Mental Disorders, Fifth Edition (DSM-5) (Regier et al., 2013), the International Classification of Sleep Disorders, Third Edition (ICSD-III) (Sateia, 2014), and the Chinese Classification and Diagnostic Criteria of Mental Disorders, Third Edition (CCMD-III) (陈彦芳, 2001).

I: The experimental group received either standalone rTMS or a combination of rTMS with additional treatments. There are no restrictions on the stimulation intensity, frequency, target area, localization method, dose, or duration.

C: Versus placebo, sham intervention, alternative therapies, or absence of rTMS intervention.

O: The study’s key results focused on various sleep metrics measured by both the Pittsburgh Sleep Quality Index (PSQI) and polysomnography (PSG). Specifically, we analyzed total sleep time (TST), sleep efficiency (SE), wake after sleep onset (WASO), sleep onset latency (SOL), rapid-eye movement (REM) latency, total arousal index (TSI), and the percentage of TST spent in each sleep stage—including N1, N2, N3, and REMS (rapid eye movement sleep). Additionally, the insomnia severity index (ISI) score, along with other sleep-related measures and adverse event records, completed our comprehensive assessment.

Other eligible criteria: We limited our analysis to English and Chinese RCTs exclusively.

### Exclusion criteria for literature

(1) Not reporting sleep-related outcomes, (2) conference abstracts, editorials, reviews, meta-analyses, theses/dissertations, case reports/series, and (3) others: unavailable full-text articles; literature where information could not be extracted; incomplete original data; unsuccessful data requests; duplicate publications.

### Data extraction

The investigators, YM and WH, independently searched the available databases to identify and retrieve scholarly works that met the established eligibility criteria. As shown in Figure 1, the study’s selection process is depicted in a flowchart, and EndNote (version X9) was used to manage all collected literature. After thoroughly reviewing the full texts, the selected articles were compiled and examined to reach a final conclusion. When incomplete data appeared in the literature, the original researchers were contacted for further details. Literature lacking usable information despite thorough efforts was not included. Disagreements were settled through discussion or by consulting CZG, an independent third reviewer. Extracted data from each study were independently organized into a pre-designed spreadsheet. The data collected from the studies included (1) sample size; (2) study characteristics (such as study design, population, intervention duration, disorder duration, comparison, TMS parameters); (3) relevant sleep outcomes, both subjective and objective; and (4) adverse effects. When available, the PSQI or PSG was selected as the sleep outcomes measure; otherwise, the most appropriate measure was used. The studies compared sham treatments with active controls and evaluated changes in overall sleep quality.

### Quality assessment

Two research specialists (Q S and S Z) independently assessed the methodological soundness of all eligible trials included in our meta-analysis, using the Cochrane Risk of Bias assessment tool (version 5.1.0). This instrument assesses six different areas to identify potential bias: (1) Random selection bias (randomization and allocation concealment); (2) Methodological Bias (Participant and Personnel blinding); (3) Outcome Assessment Bias (Blinding of Evaluation); (4) Incomplete Data Bias (Attrition Effect); (5) reporting bias (selective reporting); and (6) other potential biases. The assessment indicated that each domain was classified as low, high, or uncertain with respect to bias risk. When reviewers disagreed, they discussed the issue or consulted a third party (C-ZG) to settle their differences and come to an agreement.

### Evidence quality assessment

Two reviewers independently used the GRADE (Grading of Recommendations Assessment, Development, and Evaluation) instrument to assess evidence quality (Schünemann et al., 2008). It considers five aspects: study limitations, inconsistent results, indirect evidence, imprecision, and reporting bias. Evidence quality is categorized across four tiers: “High,” “Moderate,” “Low,” and “Extremely Low.”

### Statistical analysis

#### Data synthesis and analysis

The meta-analysis procedures were conducted with Review Manager 5.3 (Cochrane Collaboration, Oxford, UK), using weighted mean difference (WMD) or standardized mean differences (SMD) for continuous outcomes and relative risk (RR) for binary outcomes, each with a 95% confidence interval (CI) and a significance level of *α* = 0.05. The effect of TMS on sleep quality was evaluated by comparing the average change in sleep metrics—specifically, the WMD between pre- and post-treatment scores—across both the experimental and control groups. When a study reported multiple effect sizes from the same patient group, the overall impact of relevant studies was assessed using SMD. SMD allows comparison of mean differences between groups across different measurement scales in meta-analyses. We calculated separate and overall intra-group impact measures for the continuous variables. For studies in which the original data were presented as mean ± SE, SD was determined by applying the equation SE = SD/√n (n representing participant count). These data were then entered into Review Manager 5.3 for further meta-analysis. We assessed heterogeneity using the Cochrane *Q* test and the *I*^2^ statistic. When statistical heterogeneity was considered negligible (*Q* test, *p* > 0.1; *I*^2^ < 50%), we applied a fixed-effect model. However, when significant heterogeneity was present (*Q* test, *p* ≤ 0.1; *I*^2^ ≥ 50%), we adopted a random-effects model to ensure the reliability of our results. When significant heterogeneity is present, sensitivity analyses and prespecified subgroups are used to identify sources of heterogeneity and potential moderators of effect size. Data from multimodal biomarkers, such as EEG and functional connectivity, were excluded from the quantitative synthesis due to limited availability and because these results were presented graphically, making robust statistical analysis inappropriate without standardized numerical metrics.

#### Publication bias and sensitivity analysis

The meta-analysis results were presented in forest plots to visualize the distribution of the data. We used funnel plots and Egger’s regression test to evaluate publication bias, with all analyses performed in Stata 17.0 (Stata Corp, College Station, TX, USA) when a parameter was reported by ≥10 studies in the meta-analysis. We considered *p*-values < 0.05 to be significant when comparing outcome variables. The robustness of the meta-analysis findings was evaluated through sensitivity analyses, in which each study was removed individually, and studies considered high risk of bias were excluded.

### Subgroup analyses

To examine more closely the correlations between effect magnitude and these factors, and to address the considerable heterogeneity, we conducted subgroup analyses: disorder duration (6 or fewer vs. more than 6 months), treatment duration (2 or fewer vs. more than 2 weeks), stimulation site (R-DLPFC vs. L-DLPFC/DMPFC), and differences in basic treatment (rTMS combined with medication vs. medication alone; rTMS vs. other treatments).

## Results

### Search outcomes

A search produced 1,509 studies; after removing 430 duplicates, 1,079 potentially relevant records remained. Upon reviewing titles and abstracts, we excluded 583 studies, leaving 496 for full-text eligibility review. After an in-depth review of the full texts, 477 records were excluded, and 19 studies (No. 1–19) were ultimately included in this meta-analysis (Figure 1).

### Study characteristics

The results of the 19 RCTs (No. 1–19) included studies involving a total of 1,690 patients with primary sleep disorders, all without significant comorbidities. The duration of the disorder ranged from 6 to 83 months, with a median age of 44.1 years. One study (No. 10) included only older adults (>70 years), and 60% of participants were women. In these trials, the rTMS treatment duration ranged between 1 and 6 weeks. LF-rTMS was employed in all studies, with 18 utilizing 1 Hz protocols and 1 applying cTBS. Neuroimaging studies often focus on the dorsolateral prefrontal cortex (DLPFC), including the left, right, or both hemispheres. Two studies (No. 3, 13) compared rTMS with medication in the experimental and control groups. Another 4 (No. 2, 4, 5, 16) compared combined rTMS and medication against pharmacotherapy alone. One study assessed the efficacy of rTMS combined with acupuncture versus acupuncture alone. Eight studies (No. 6, 7, 10, 11, 12, 14, 18, 19) evaluated rTMS compared to placebo control. Medications routinely administered alongside rTMS included lorazepam, tranquillizer, estazolam, and zolpidem. Objective sleep assessment was achieved through PSG, sleep diaries, and EEG recordings. Among subjective sleep measures, the PSQI was the most commonly used, followed by the ISI Score. Most studies assessed outcomes shortly after the intervention, with 5 studies (No. 11, 15, 16, 17, 18) also collecting data at 2–24 weeks of follow-up. Table 1 outlines the specific traits of individual studies.

**Table 1:** Characteristics of the 19 included studies, which encompassed 23 trials.

Study name	Sample size (I/C)	Mean age (years) (I/C)	Gender (M/F)	Diagnosis criteria	Disorder duration	Subtypes of insomnia	Intervention		Treatment duration (w)	Drug, intervention	Adverse effects	Main cognitive outcome measure
							Intervention	Control				
1. 刘雅妮 et al. (2024)	(16/15)	(44.6/36.7)	(5/26)	ICSD-III	NR	Primary	1 Hz, Right DLPFC	CBT-I	6 weeks (30 times)	NR	NR	PSQI (total score), ISI, PSG
2. 林 et al. (2022)	(80/80)	(47.5/45.3)	(82/78)	ICD-10	(25.5/23.3) (w)	Primary	1 Hz, Right DLPFC + Medication	Medication	4 weeks (20 times)	Lorazepam	mild headache; dizziness; nausea	PSQI (total score)
3. 张春华 et al. (2013)	(30/30)	(41.7/40.4)	(27/33)	CCMD-3	(7.0/8.7) (m)	Primary	1 Hz, Bilateral frontal and parietal areas	Medication	2 weeks (14 times)	Tranquillizer	NR	PSG
4. 陈妙 et al. (2017)	(60/60)	(50.1/51.3)	(48/72)	DSM-IV	(10.9/11.1) (m)	Primary	1 Hz, Right DLPFC+Medication	Medication	2 weeks (10 times)	Zolpidem tartrate	mild headache; nausea; mild dizziness	PSQI (total score, seven subscales)
5. 陈妙 et al. (2017)	(50/50)	(39.6/41.3)	(53/47)	ICD-11	NR	Primary	1 Hz, Left DLPFC+Medication	Medication	3 weeks (15 times)	Trazodone	NR	PSQI (total score)
6. 冯秀娟 et al. (2017)	(43/37)	(44.1/45.1)	(26/54)	ICD-10	(8.8/7.6) (m)	Primary	1 Hz, Right DLPFC	Sham	2 weeks (10 times)	NR	Headache	PSQI (total score, seven subscales)
7. 陆艺 et al. (2023)	a. (60/60) b. (60/60) c. (60/60)	(45.2/43.2) (44.2/43.2) (45.1/43.2)	(49/71) (46/74) (47/73)	ICD-10	(6.1/6.1) (m) (6.0/6.1) (m) (6.1/6.1) (m)	Primary	1 Hz, Right DLPFC 1 Hz, Left DLPFC 1 Hz, Cz	Sham	4 weeks (20 times)	NR	NR	PSQI (total score)
8. 瞿砚舟 et al. (2020)	(32/28)	(37.9/37.7)	(21/29)	ICD-10	NR	Primary	1 Hz, DLPFC +TPO	cTBS, DLPFC + TPO	2 weeks (10 times)	NR	NR	PSQI (total score)
9. 陶晟 et al. (2020)	(35/35)	(44.2/43.1)	(41/29)	ICD-10	(7.9/8.2) (m)	Primary	1 Hz, Right DLPFC	AMFT	4 weeks (20 times)	NR	NR	PSQI (total score), Endocrine level (cortisol, glutamate, glycine)
10. 张春华 et al. (2013)	(71/72)	(72.6/73.1)	(51/92)	ICSD-III	(18.2/19.1) (m)	Primary	1 Hz, Right DLPFC	Sham	4 weeks (20 times)	Estazolam	Dizziness; mild headache; Drowsiness and fatigue	PSQI (total score), EEG parameters, Serum 5-HT and NPY levels, PSG
11. Chen et al. (2025)	(15/14)	(20.2/19.8)	(13/16)	ICSD-III	NR	Primary	1 Hz, Left DLPFC	Sham	2 weeks (10 times)	NR	mild headache;	PSQI (total score), ISI
12. Gong et al. (2025)	(22/22)	(32.8/33.2)	(7/37)	ICSD-III	(6.0/5.7) (Y)	Primary	1 Hz, Right DLPFC	Waitlist	4 weeks (20 times)	NR	Drowsiness; mild headache;	PSQI (total score), ISI
13. Jiang et al. (2013)	d. (45/45) e. (45/45)	(48.3/48.1) (48.3/47.0)	(40/50) (41/49)	DSM-IV	(11.2/12.5) (m) (11.2/10.2) (m)	Primary	1 Hz, Right DLPFC	Medication; Psychotherapy	2 weeks (10 times)	Estazolam	NR	PSQI (total score), PSG
14. Lin et al. (2023)	(36/13)	(53.5/37.0)	(11/38)	DSM-V	NR	Primary	1 Hz, Left DMPFC	Sham	2 weeks (10 times)	NR	NR	PSQI (total score), PSG
15. Zhang et al. (2018)	(40/38)	(51.3/49.8)	(11/67)	DSM-V	(12.7/13.0) (Y)	Primary	1 Hz, Left PFC + acupuncture	Sham + acupuncture	4 weeks (12 times)	NR	NR	PSQI (total score), ISI
16. Zheng et al. (2025)	(19/23)	(45.9/47.0)	N	DSM-V	(6.5/6.9) (Y)	Primary	1 Hz, Left DLPFC+Medication	Medication	4 weeks (16 times)	Dexmedetomidine	Dizziness; Local pain; Thirst; Hypertension; Bradycardia	PSQI (total score)
17. Zhou et al. (2024)	f. (50/51) g. (50/56)	(47.0/42.4) (47.0/44.5)	(32/69) (38/68)	ICD-11	(79.1/83.4) (m) (79.1/75.8) (m)	Primary	1 Hz, Right DLPFC	1 Hz, Right DLPFC (tDCS) 1 Hz; Right DLPFC (tDCS+rTMS)	4 weeks (20 times)	Zopiclone	transient headache; mild skin redness	PSQI (total score, seven subscales)
18. Zhu et al. (2024)	(21/25)	(38.0/42.4)	(14/32)	DSM-IV	(56.1/56.3) (m)	Primary	cTBS, Right DLPFC	Sham	2 weeks (10 times)	NR	Mild headache; drowsiness	PSQI (total score), PSG, ISI
19. Shao et al. (2024)	(22/24)	(44.5/46.6)	(20/26)	DSM-V	NR	Primary	1 Hz, Left DLPFC	Sham	1 weeks (5 times)	NR	NR	PSQI (total score), ISI

a, Right DLPFC group (rTMS) vs Sham group; b, Left DLPFC group (rTMS) vs Sham group; c, Cz group (rTMS) vs Sham group; d, Right DLPFC group (rTMS) vs Medication group; e, Right DLPFC group (rTMS) vs Psychotherapy group; f, Right DLPFC group (rTMS) vs Right DLPFC group (tDCS); g, Right DLPFC group (rTMS) vs Right DLPFC group (tDCS+rTMS); rTMS, repetitive transcranial magnetic stimulation; tDCS, transcranial direct current stimulation; AMFT, Alternating magnetic field therapy; cTBS, Continuous theta-burst stimulation; DLPFC, dorsolateral prefrontal cortex; PFC, prefrontal cortex; CBT-I, cognitive behavioral therapy for insomnia; DMPFC, dorsal medial prefrontal cortex; TPO, temporoparietal-occipital; M, man; F, female; I, Intervention; C, Control; NR, Not Reported; m, month; w, week; PSQI, Pittsburgh Sleep Quality Index; PSG, polysomnography; ISI, insomnia severity index; EEG, electroencephalogram; 5-HT, hoxytryptamine; NPY, neuropeptide.

### Adverse events

Of the 23 qualified studies, 50 patients in the six project studies (No. 6, 10, 11, 12, 17, 18) reported adverse effects related to rTMS, including mild headache, dizziness, drowsiness, and fatigue. Besides the rTMS group, 30 patients in the three studies (No. 2, 4, 16) reported adverse drug reactions such as nausea, mild dizziness, thirst, hypertension, and bradycardia. Compared with drug treatment, the adverse reactions of magnetic stimulation seem to be milder, while those caused by drugs are more severe. The side effects of TMS were transient and resolved quickly after the procedure. TMS is regarded as safe and effective (Cotovio et al., 2023).

### Risk of bias assessment

**Figure 2:**
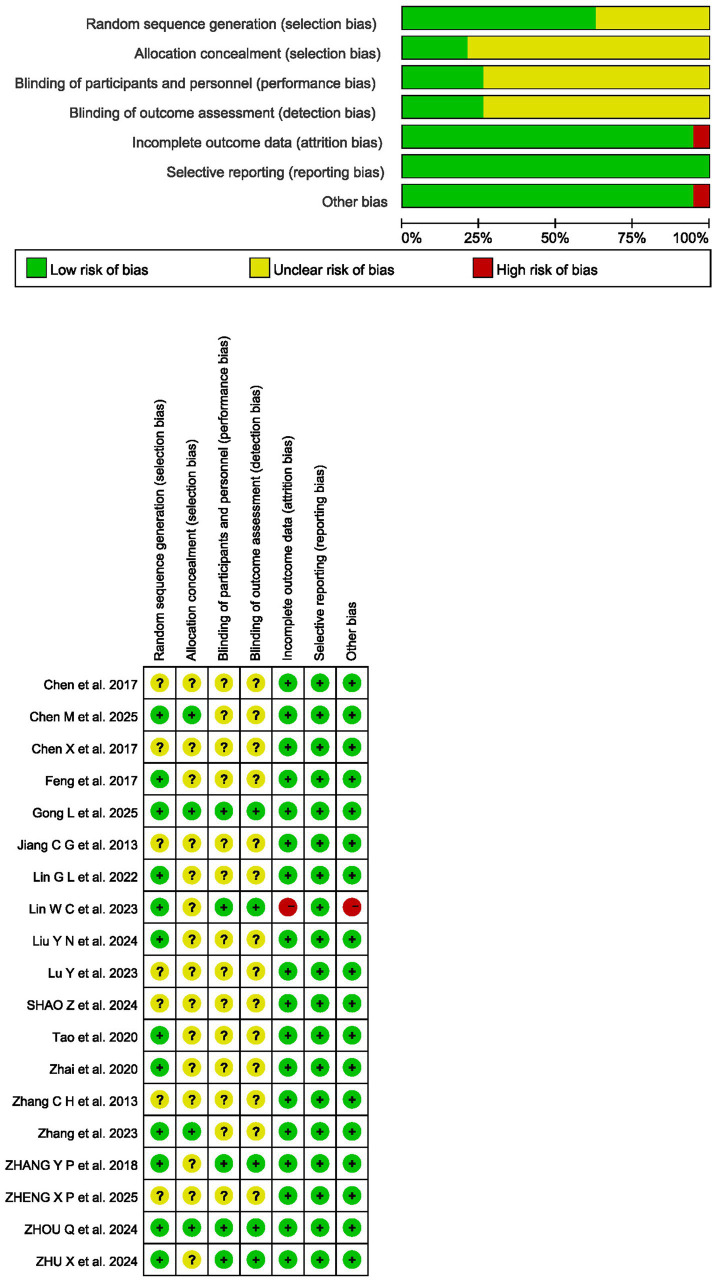
The risk of bias of included studies based on the Cochrane’s handbook. Risk of bias summary according to the Cochrane risk of bias tool: “+,” “−,” and “?” respectively indicate low, high, and unclear risk of bias.

Two researchers (Q S and S Z) assessed potential bias in the 19 selected studies using the Cochrane Handbook criteria. Among the studies included, 12 (Nos. 1, 2, 6, 8, 9, 10, 11, 12, 14, 15, 17, 18) produced randomized sequences using low-risky distribution techniques, including random number generators, coin tossing, and computer-generated random number sequences. Seven studies did not specify their randomization method, resulting in an unclear risk assessment. Four studies (No. 10, 11, 12, 17) reported allocation concealment. Five studies (No. 12, 14, 15, 17, 18) reported blinding of participants, staff, and evaluators; both aspects were rated as low risk, and the remaining studies were assessed as unclear risk. Lin et al. (2023) were judged to be at high risk due to incomplete outcome data, while other risks of bias were considered high due to an imbalance in group allocation. Regarding selective outcome reporting, 23 studies were judged to be at low risk of bias because they reported all outcomes. Of the 19 studies reviewed, 11 were rated low risk, 7 were rated unclear, and 1 was rated high risk. Figure 2 summarizes the quality assessment.

### Evidence quality evaluation results

The quality of evidence for the 14 outcomes assessed across the 19 included studies was evaluated using the GRADE framework (Table 2). According to this assessment, six outcomes (42.86%) are supported by moderate-quality evidence, four outcomes (28.57%) by low-quality evidence, and four outcomes (28.57%) by very low-quality evidence. This results in a moderate overall level of evidence. The low ratings for some outcomes mainly stem from several limitations. Key concerns include the risk of methodological bias (inadequate randomization or blinding), imprecision in estimates due to small sample sizes and variability across studies, and substantial statistical heterogeneity that undermines the reliability of the combined results.

**Table 2:** Results of evidence quality.

Certainty assessment							No of patients		Effect		Certainty	Importance
No. of studies	Study design	Risk of bias	Inconsistency	Indirectness	Imprecision	Other considerations	[Real rTMS]	[Sham rTMS]	Relative (95% CI)	Absolute (95% CI)		
PSQI total scores												
8	Randomized trials	Serious^a^	Not serious	Not serious	Not serious^a^	None	410	387	–	MD 3 lower (3.83 lower to 2.17 lower)	⨁⨁⨁◯ Moderate^a^	Critical
PSG (SE)												
3	Randomized trials	Serious^a^	Not serious	Not serious	Very serious^b^	None	128	110	–	MD 3.19 higher (1.09 higher to 5.3 higher)	⨁◯◯◯ Very low^a,b^	Not important
PSG (TST)												
2	Randomized trials	Serious^a^	Not serious	Not serious	Very serious^b^	None	57	38	–	MD 19.71 lower (69.36 higher to 29.94 higher)	⨁◯◯◯ Very low^a,b^	Not important
PSG (SL)												
2	Randomized trials	Serious^a^	Not serious	Not serious	Serious^c^	None	107	85	–	MD 2.12 higher (12.72 lower to 16.96 higher)	⨁⨁◯◯ Low^a,c^	Not important
PSG (WASO)												
2	Randomized trials	Serious^a^	Not serious	Not serious	Very serious^b^	None	57	38	–	MD 6.5 higher (11.6 lower to 24.6 higher)	⨁◯◯◯ Very low^a,b^	Not important
PSG (REMS)												
2	Randomized trials	Serious^a^	Not serious	Not serious	Very serious^b^	None	92	97	–	MD 3.87 higher (0.68 lower to 8.42 higher)	⨁◯◯◯ Very low^a,b^	Not important
ISI scores												
3	Randomized trials	Serious^a^	Not serious	Not serious	Serious^c^	None	58	63	–	MD 4.59 lower (6.38 lower to 3.51 lower)	⨁⨁◯◯ Low^a,c^	Important
PSQI total scores (Disorder duration)												
5	Randomized trials	Serious^a^	Not serious	Not serious	Not serious	None	337	336	–	MD 3.1 lower (4.09 lower to 2.11 lower)	⨁⨁⨁◯ Moderate^a^	Important
PSQI total scores (Treatment duration)												
8	Randomized trials	Serious^a^	Not serious	Not serious	Not serious	None	410	387	–	MD 3 lower (3.83 lower to 2.17 lower)	⨁⨁⨁◯ Moderate^a^	Important
ISI scores (Treatment duration)												
4	Randomized trials	Serious^a^	Not serious	Not serious	Serious^c^	None	80	85	–	MD 5.05 lower (6.33 lower to 3.78 lower)	⨁⨁◯◯ Low^a,c^	Not important
PSQI total scores (Stimulation site)												
9	Randomized trials	Serious^a^	Not serious	Not serious	Not serious	None	350	327	–	MD 3.25 lower (4.02 lower to 2.48 lower)	⨁⨁⨁◯ Moderate^a^	Important
ISI total scores (Stimulation site)												
4	Randomized trials	Not serious	Not serious	Not serious	Serious^c^	None	80	85	–	MD 5.05 lower (6.33 lower to 3.78 lower)	⨁⨁⨁◯ Moderate^c^	Important
PSQI total scores (rTMS vs other treatment)												
5	Randomized trials	Serious^a^	Not serious	Not serious	Serious^c^	None	178	173	–	MD 2.13 lower (4.29 lower to 0.02 higher)	⨁⨁◯◯ Low^a,c^	Not important
PSQI total scores (rTMS + medication vs medication)												
4	Randomized trials	Serious^a^	Not serious	Not serious	Not serious	None	209	213	–	MD 2.77 lower (4.62 lower to 0.92 lower)	⨁⨁⨁◯ Moderate^a^	Important

CI: confidence interval; MD: mean difference; PSQI: Pittsburgh Sleep Quality Index; PSG: polysomnography; SE: sleep efficiency; TST: total sleep time; SL: sleep latency; WASO: wake after sleep onset; REMS: rapid eye movement sleep; ISI: insomnia severity index.

Explanations: ^a^(2/3) The included studies have a risk of bias in terms of randomization, allocation concealment, and blinding. ^b^The sample size is too small, and the confidence interval is relatively wide. ^c^The sample size included in the study was less than 400.

### Effects of rTMS on insomnia disorder (real rTMS versus sham rTMS) (No. 6, 7, 10, 11, 12, 14, 18, 19)

#### PSQI total scores and the seven subscales of the PSQI

**Figure 3:**
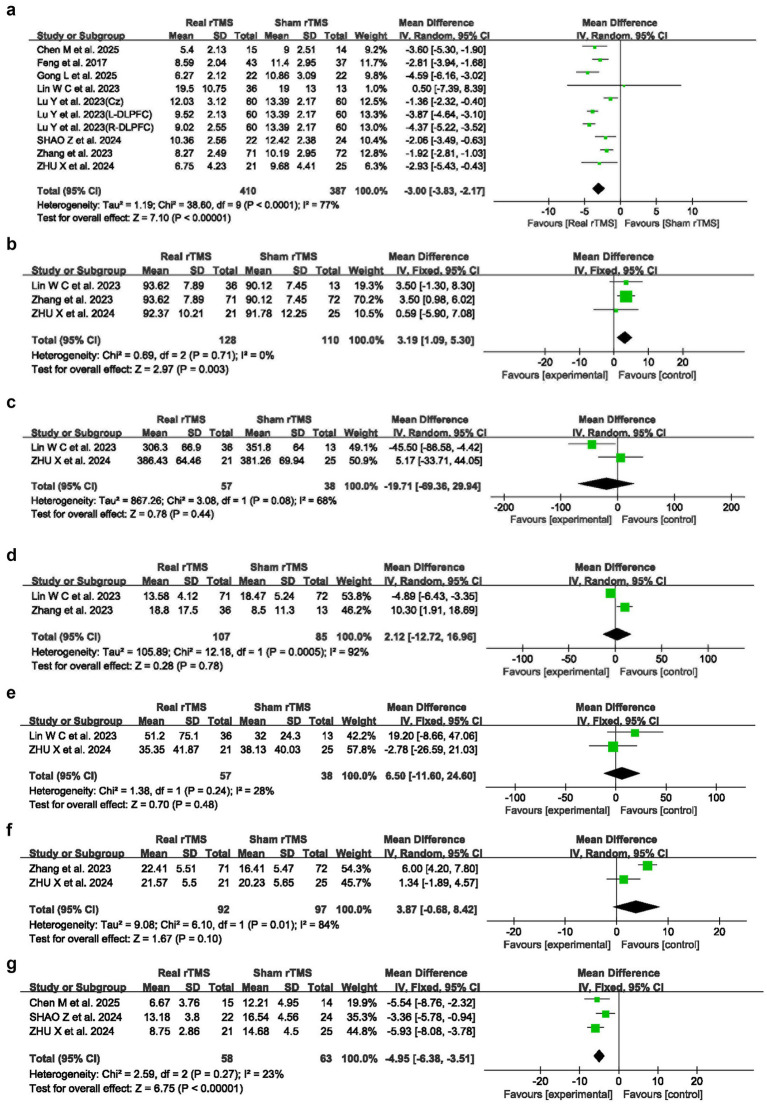
Forest plot: meta-analysis of rTMS on different sleep outcomes **(a–g)** (Real rTMS vs. Sham rTMS).

**Figure 4:**
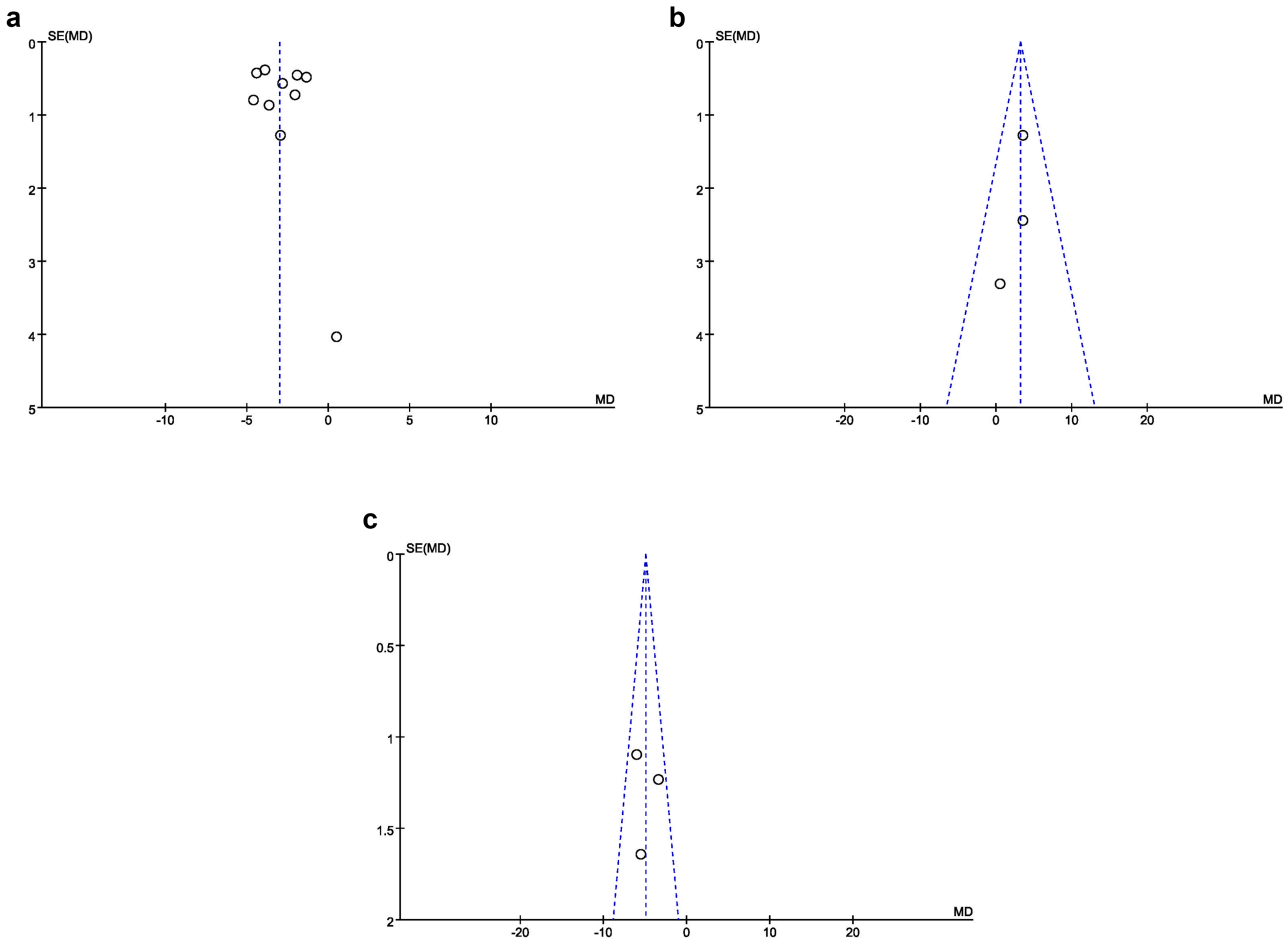
Funnel plot: rTMS on different sleep outcomes **(a–c)**.

A total of 8 studies and 10 trials involving 797 patients were selected to assess the effects of rTMS on PSQI total scores. The meta-analysis revealed that rTMS significantly improved sleep quality relative to sham treatment (WMD: −3.00; 95% CI: −3.83, −2.17; *p* < 0.0001). In terms of the absolute difference, this reflects an average 3-point decrease on the PSQI, and significant heterogeneity was observed (*I*^2^ = 77%, *p* < 0.0001) (Figure 3a). Thus, a sensitivity analysis was performed. The trial analysis revealed that after excluding the highly weighted study (陆艺 et al., 2023), the *I*^2^ statistic among the remaining studies was 46% (*p* = 0.09). Moreover, the disparity between the two groups remained statistically significant (WMD: −2.82; 95% CI: −3.63, −2.01; *p* < 0.0001). The overall pooled estimate of the total PSQI score was considered robust. High heterogeneity is linked to both the stimulation site and disease duration in the study by Lu et al. Further exploration of these factors will be conducted in the subgroup analysis. The funnel plot’s visual inspection indicated no tendency toward publication bias (Figure 4a). Further analysis using Egger’s regression showed no significant asymmetry (*p* = 0.83), suggesting that our results are robust and reliable.

Four trials reported scores for the seven PSQI subscales. However, compared with the Sham rTMS group, only one study provided scores for all seven subscales. As a result, a data summary analysis could not be conducted, and descriptive statistics were used instead. The descriptive analysis indicated that sleep parameters, including Sleep Quality (SQ), Sleep Latency (SL), Sleep Time (ST), and Daytime Dysfunction (DS), were significantly improved in the rTMS cohort compared with the placebo group (*p* < 0.05). Nonetheless, Sleep Efficiency (SE), Sleep Disturbance (SD), and Hypnotic Use (HU) did not show significant improvements (*p* > 0.05).

#### PSG

Two studies compared sleep onset latency (SOL), wake after sleep onset (WASO), total sleep time (TST), and rapid eye movement sleep (REMS) between the rTMS and sham groups. Meanwhile, three studies assessed sleep efficiency (SE). The collective findings demonstrated rTMS surpassed sham treatment in enhancing SE (fixed-effects model: WMD 3.19; 95% CI 1.09, 5.30; *p* = 0.003) (Figure 3b). Additionally, the funnel plot showed no significant asymmetry (Figure 4b). The rTMS group showed no meaningful improvement in TST (random-effects model: WMD − 19.71; 95% CI − 69.36, 20.94; *p* = 0.44), SOL (random-effects model: WMD 2.12; 95% CI − 12.72, 16.96; *p* = 0.78), WASO (fixed-effects model: WMD 6.50; 95% CI − 11.60, 24.60; *p* = 0.48), and REMS (random-effects model: WMD 3.87; 95% CI −0.68, 8.42; *p* = 0.10) compared with sham treatment (Figures 3c–f). Nonetheless, the combined data for TST, SL, and REMS showed considerable heterogeneity, with *I*^2^ values ranging from 68 to 92%. Due to the limited comparative studies, sensitivity analysis was not conducted (*n* = 2). The observed heterogeneity may be attributable to unequal sample sizes (rTMS group = 36 vs. Sham group = 13; Lin et al., 2023) and the limited number of included studies. Table 3 and Figures 3b–f show PSG and actigraphy results for TST, SL, SE, WASO, and REMS in rTMS meta-analyses.

**Table 3:** Meta-analysis of PSG of rTMS vs. Sham rTMS.

Domain	*n*	No. of participants	MD [95%CI]	*I*^2^ (%)	*p* Value
TST	2	95	−19.71 [−69.36, 20.94]	68	0.44
SOL	2	192	2.12 [−12.72, 16.96]	92	0.78
SE	3	238	3.19 [1.09, 5.30]	0	0.003
WASO	2	95	6.50 [−11.60, 24.60]	28	0.48
REMS	2	189	3.87 [−0.68, 8.42]	84	0.10

MD, mean difference; CI, confidence interval; TST, total sleep time; SOL, sleep onset latency; SE, sleep efficiency; WASO, wake after sleep onset; REMS, rapid eye movement sleep.

#### ISI

Three studies (No. 11, 18, 19) involving 121 participants (58 in the Real rTMS cohort and 63 in the placebo rTMS cohort) evaluated the effects of rTMS using the ISI. The real rTMS group showed a statistically significant effect compared with the Sham rTMS group (fixed-effects model: WMD − 4.95; 95% CI − 6.38, −3.51; *p* < 0.001) minimal heterogeneity noted (*I*^2^ = 23%, *p* = 0.27) (Figure 3g). Additionally, the funnel plot showed no significant asymmetry (Figure 4c).

### Subgroup analyses

Tables 4, 5 outline our subgroup analyses, which investigated how different related factors influence clinical outcomes.

**Figure 5:**
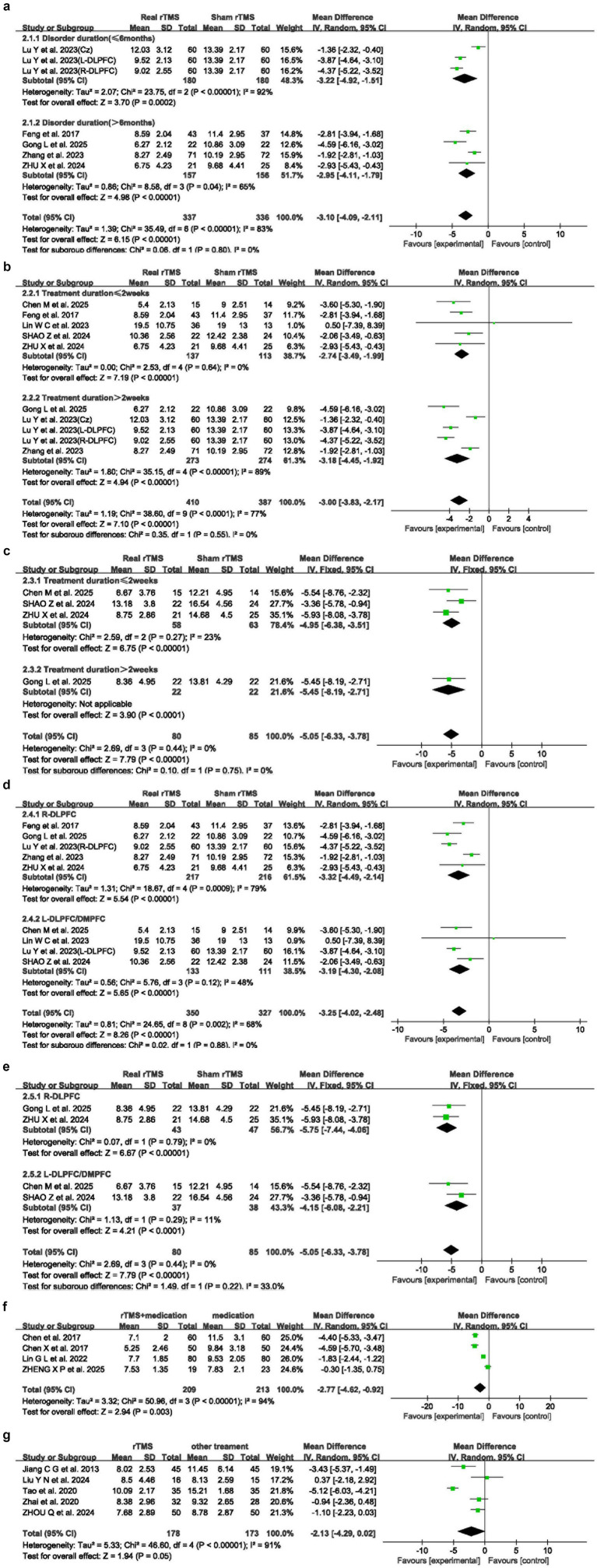
Forest plot: subgroup analyses of rTMS on sleep outcomes **(a–g)**.

**Figure 6:**
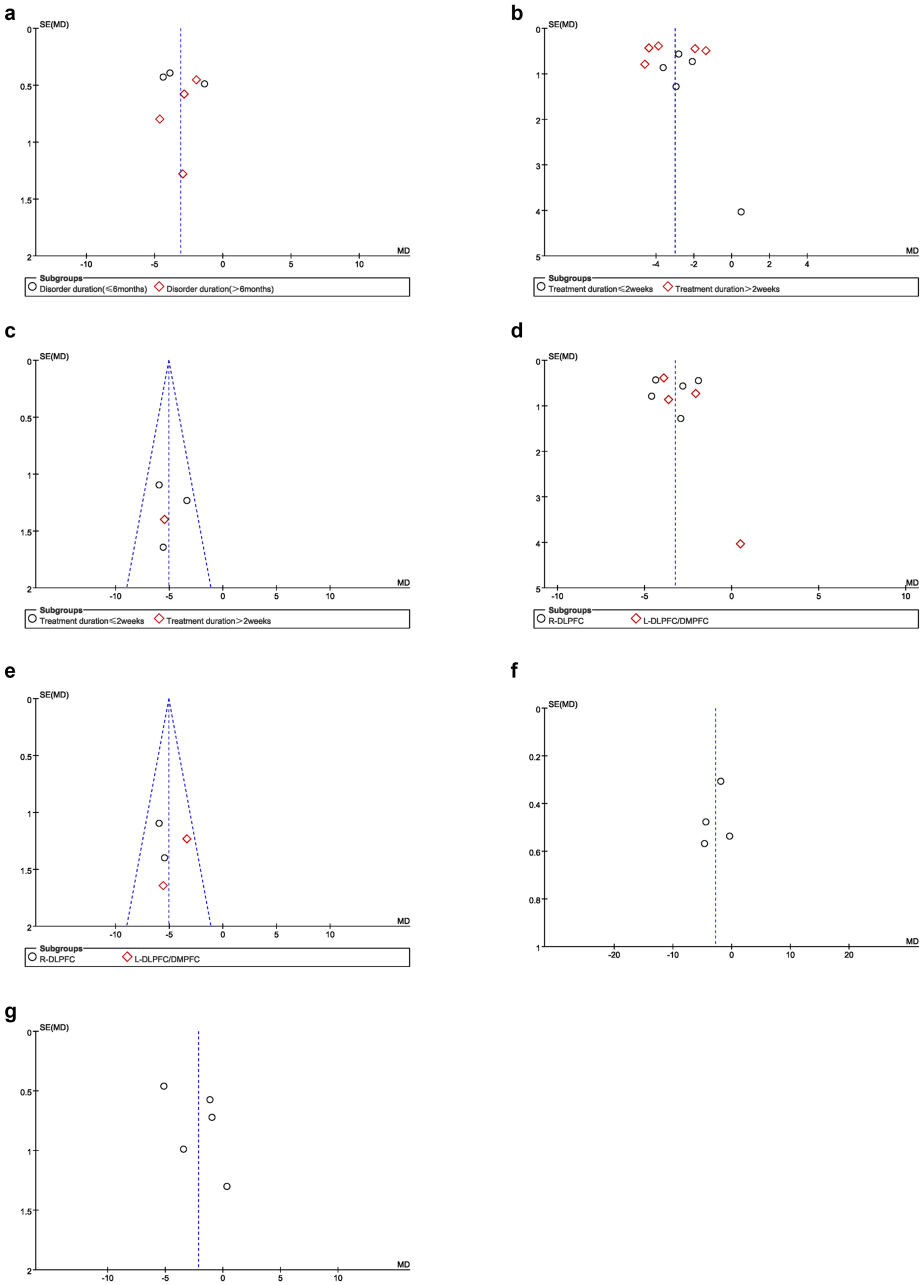
Funnel plot: subgroup analyses of rTMS on sleep outcomes **(a–g)**.

1. Disorder duration (real rTMS versus sham rTMS) (No. 6, 7, 10, 12, 18)
  Subgroup analysis based on sleep disorder duration (ranging from 6 months to 83 months, with 6 months as the cutoff): those with more than 6 months and those with 6 months or less. A total of 13 studies, encompassing 17 trials, reported the disease duration in patients with primary insomnia. However, only 7 of these trials directly compared real rTMS against sham rTMS. Four trials reported patients with disorder durations of over 6 months, while three trials included patients with durations of 6 months or less. Findings indicated rTMS enhanced PSQI overall scores for both participant cohorts, with the effect seeming even greater in patients with less than 6 months of ID (WMD: −3.22, 95% CI: −4.92, −1.51; *p* < 0.001) (Figure 5a). Given the significant heterogeneity, removing each trial sequentially did not alter the overall results. When 陆艺 et al. (2023) and 张雷鸣 et al. (2023) were excluded, heterogeneity was significantly reduced, likely due to the new stimulation site (Cz) in the study and the group’s maximum age (73.15 ± 7.22 years). Additionally, the funnel plot showed no significant asymmetry (Figure 6a).
2. Treatment duration (real rTMS versus sham rTMS) (No. 6, 7, 10, 11, 12, 14, 18, 19)
  2.1. PSQI total scores. rTMS treatment durations across studies ranged from 2 to 6 weeks, with 2 weeks identified as pivotal. Both treatment courses longer than 2 weeks (WMD, −3.18; 95% CI, −4.45, −1.92; *p* < 0.0001) and those of 2 weeks or less (WMD, −2.74; 95% CI, −3.49, −1.99; *p* < 0.0001) showed significant improvements in PSQI scores (Figure 5b). This trend of improvement appears more pronounced among ID patients with treatment durations longer than 2 weeks. Despite substantial heterogeneity, removing each trial sequentially did not alter the overall results. When 陆艺 et al. (2023) and 张雷鸣 et al. (2023) were excluded, heterogeneity decreased significantly, likely due to the new stimulation site (Cz) in those studies and the group’s maximum age (73.15 ± 7.22 years). Additionally, the funnel plot showed no significant asymmetry (Figure 6b).
  2.2. ISI scores. Four trials reported ISI score-related indicators, with patients having a treatment duration of >2 weeks (WMD, −5.45; 95% CI −8.19, −2.71; *p* < 0.0001) and those with ≤2 weeks of treatment duration (WMD, −4.95; 95% CI −6.38, −3.51; *p* < 0.0001) showing significant improvements in ISI scores in both groups (Figure 5c). Additionally, the funnel plot showed no significant asymmetry (Figure 6c).
3. Stimulation site (real rTMS versus sham rTMS) (No. 1, 2, 3, 4, 5)
  1. 3.1. PSQI total scores. Analysis by stimulation site revealed that the improvement in PSQI scores was significant for both the L-DLPFC/DMPFC (WMD, −3.19; 95% CI −4.30, −2.08; *p* < 0.001) and the R-DLPFC (WMD, −3.32; 95% CI −4.49, −2.14; *p* < 0.001), with no discernible difference between the two groups (Figure 5d). Moderate heterogeneity was observed in the R-DLPFC group; removing each trial individually did not alter the overall results. When 张雷鸣 et al. (2023) were excluded, heterogeneity decreased significantly, likely due to the group’s maximum age of 73.15 ± 7.22 years. The funnel plot showed no significant asymmetry (Figure 6d).
  2. 3.2. ISI scores. Four trials reported indicators related to ISI scores: the R-DLPFC group (WMD, −5.75; 95% CI −7.44, −4.06; *p* < 0.0001) and the L-DLPFC/DMPFC group (WMD, −4.15; 95% CI −6.08, −2.21; *p* < 0.0001). The ISI scores of both groups were significantly improved (Figure 5e). This improvement trend appears more pronounced in the R-DLPFC group. No significant heterogeneity was observed. Furthermore, the funnel plot shows no obvious asymmetry (Figure 6e).
4. Differences in basic treatment
  1. 4.1 Analysis of subgroup A (rTMS combined with medication versus medication alone) included 4 trials with 422 patients to evaluate the effects on the overall PSQI score. The meta-analysis revealed that adding rTMS to medication treatment resulted in a statistically significant improvement in total PSQI scores compared with medication alone. (WMD: −2.77; 95% CI: −4.62, −0.92; *p* = 0.03). Notable heterogeneity was detected (*I*^2^ = 94%, *p* < 0.0001) (Figure 5f). Consequently, a sensitivity assessment was conducted. The analysis showed that after excluding the highly weighted study (Lin et al., 2023; Zheng et al., 2025), the I^2^ statistic among the remaining studies was 0% (*p* = 0.80). Additionally, the groups’ divergence was statistically significant (WMD: −4.48; 95% CI: −5.91, −3.76; *p* < 0.0001). High heterogeneity may be due to differences in the types of medication used across research groups. The funnel plot’s visual inspection indicated no tendency toward publication bias (Figure 6f).
5. In the analysis of subgroup B (rTMS versus other treatments)
  Patients who received rTMS showed no significant improvement in PSQI scores compared with other treatment cohorts. (WMD, −2.13; 95% CI: −4.29, 0.02; *p* = 0.05). High heterogeneity was observed (*I*^2^ = 91%, *p* < 0.0001) (Figure 5g). Consequently, we performed a sensitivity analysis. The results indicate that excluding high-weight studies (陶晟 et al., 2020) reduces the *I*^2^ statistic to 55% (*p* = 0.08). Moreover, the groups differed significantly (WMD, −1.33; 95% CI: −2.56, −0.10; *p* = 0.03). Following treatment, the rTMS group demonstrated a markedly greater reduction in total PSQI scores than other treatment approaches. The funnel plot revealed no evidence of publication bias affecting results (Figure 6g).

**Table 4:** Subgroup analysis for PSQI total scores and ISI scores of Real rTMS vs. Sham rTMS.

Subgroup	No. trials	Sample size (Real/Sham)	MD (95% CI)	Heterogeneity (*I*^2^) (%)	*p* value
Disorder duration (months) (PSQI total scores)					
≤6	3	(180/180)	−3.22 [−4.92, −1.51]	92	<0.001
>6	4	(157/156)	−2.95 [−4.11, −1.79]	65	0.04
Treatment duration (weeks) (PSQI total scores)					
≤2	5	(137/113)	−2.74 [−3.49, −1.99]	0	<0.001
>2	5	(273/274)	−3.18 [−4.45, −1.92]	89	<0.001
Treatment duration (weeks) (ISI scores)					
≤2	3	(58/63)	−4.95 [−6.38, −3.51]	23	<0.001
>2	1	(22/22)	−5.45 [−8.19, −2.71]	0	<0.001
Stimulation site (PSQI total scores)					
R-DLPFC	5	(217/216)	−3.32 [−4.49, −2.14]	79	<0.001
L-DLPFC/DMPFC	4	(133/111)	−3.19 [−4.30, −2.08]	48	<0.001
Stimulation site (ISI scores)					
R-DLPFC	2	(43/47)	−5.75 [−7.44, −4.06]	0	<0.001
L-DLPFC/DMPFC	2	(37/38)	−4.15 [−6.08, −2.21]	11	<0.001

MD, mean difference; CI, confidence interval; rTMS, Repetitive transcranial magnetic stimulation; L-DLPFC, left dorsolateral prefrontal cortex; L-DMPFC, left dorsal medial prefrontal cortex; PSQI, Pittsburgh Sleep Quality Index; ISI, insomnia severity index.

**Table 5:** Subgroup analysis for PSQI total scores of rTMS + medication vs. medication and rTMS vs. other treatments.

Subgroup	No. trials	Sample size (Real/Sham)	MD (95% CI)	Heterogeneity *(I^2^) (%)*	*p* value
Differences in basic treatment (PSQI total scores)					
rTMS + medication	4	(209)	−2.77 [−4.62, −0.92]	94	0.003
medication	4	(213)			
Differences in basic treatment (PSQI total scores)					
rTMS	5	(178)	−2.13 [−4.29, 0.02]	91	0.05
other treatments	5	(173)			

MD, mean difference; CI, confidence interval; rTMS, Repetitive transcranial magnetic stimulation; PSQI, Pittsburgh Sleep Quality Index.

### Publication bias and sensitivity analysis

**Figure 7:**
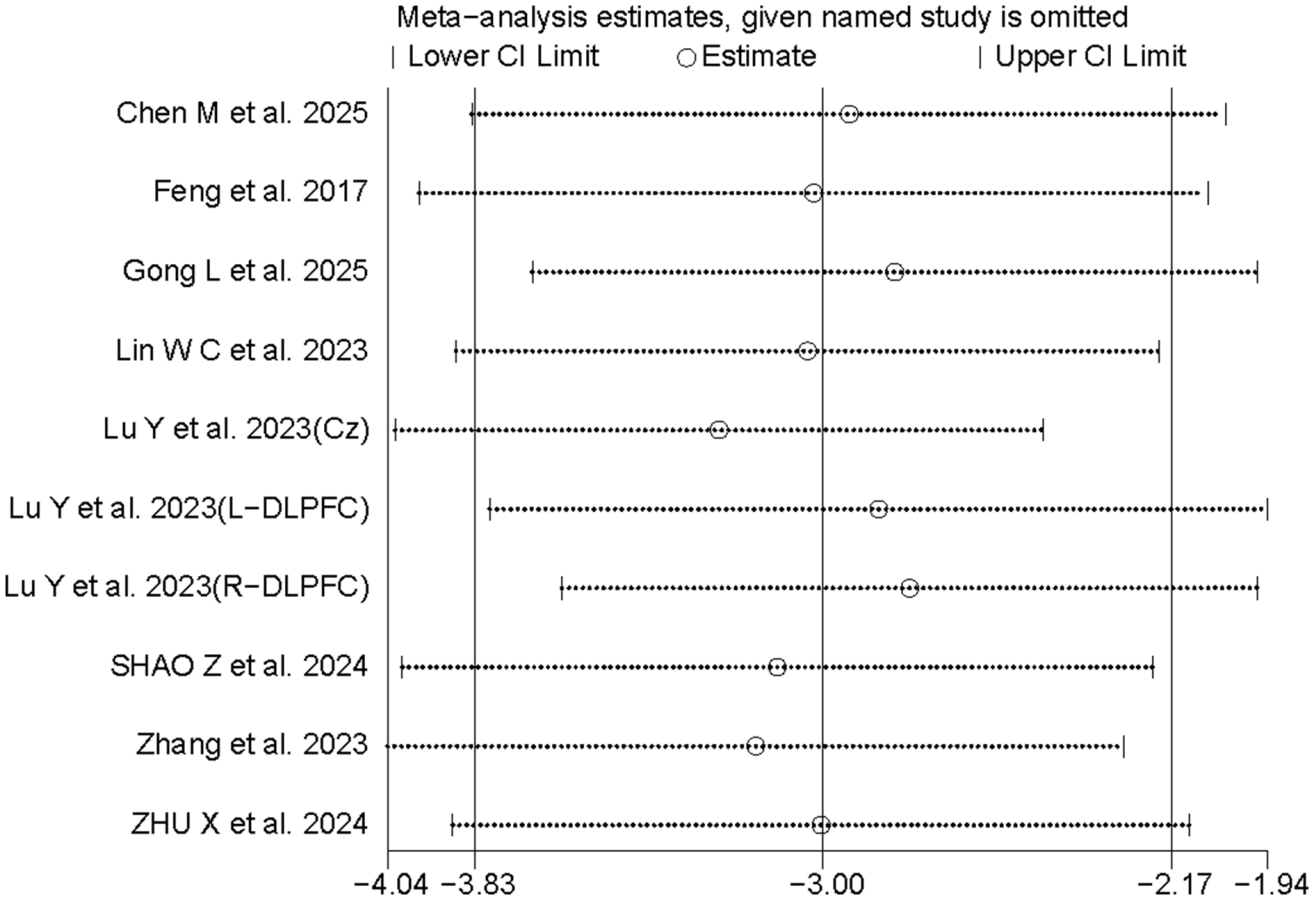
Sensitivity analysis of rTMS on PSQI total scores.

All eligible studies were assessed for publication bias. (Figures 4, 6) shows a symmetrical funnel plot, indicating a balanced spread of studies. Additionally, Egger’s test assessed publication bias among the real rTMS group with respect to overall PSQI scores. The results indicated no significant publication bias (*p* = 0.83). The robustness of the primary findings was confirmed by sensitivity analysis, which showed no significant changes in the pooled estimates (Figure 7). This analysis further identified that heterogeneity in parameters such as PSQI and ISI was likely driven by clinical factors, including older age, stimulation site, and background hypnotic use, and that this heterogeneity diminished after excluding studies with these characteristics.

## Results-based discussion

We systematically searched RCTs on rTMS for ID treatment. This meta-analysis included 19 studies with 1,690 participants and used a detailed meta-analytic process to assess the therapeutic effect of rTMS on ID. The primary outcomes identified were as follows: (1) When we compared the two groups, the participants receiving real rTMS treatment showed significantly better sleep quality than those in the sham rTMS group, as reflected in both the PSQI total score and ISI measurements; (2) Additionally, Objective metrics, including PSG parameters (SE; TST; SL; WASO; REMS), revealed significant findings. Compared with the sham group, the rTMS intervention significantly improved sleep quality, as indicated by PSG (SE). Subgroup analyses indicated: (1) Compared to medication alone, rTMS combined with medication or used as an adjunct demonstrated superior efficacy in sleep improvement. Moreover, after excluding the high-heterogeneity study by 陶晟 et al. (2020), the rTMS group still showed a statistically significant improvement in sleep compared with other treatments [CBT-I, AMFT (alternating magnetic field therapy), Psychotherapy, and tDCS (transcranial direct current stimulation)]. (2) Although rTMS significantly improved sleep quality regardless of disorder duration (>6 vs. ≤6 months) or treatment length (>2 vs. ≤2 weeks)—with no significant differences between subgroups—a non-significant trend suggested greater improvement in patients with a shorter disorder duration (<6 months) and a longer treatment course (≥2 weeks), indicating a need for further focused research. (3) Eleven studies used the R-DLPFC as the stimulation target. Other studies targeted alternative regions, including the L-DLPFC and the DMPFC. Although both subgroups showed similar improvements in sleep, the findings did not reveal any statistically significant variation among them. Since R-DLPFC was the most frequently used target and represents a main consensus in the literature, further research in this area is justified. However, therapeutic effectiveness is not the only factor that determines clinical usefulness in practice. Risks of adverse effects and serious events are also important considerations. Medications often cause adverse reactions, including lethargy, exhaustion, high blood pressure, slow heart rate, and vomiting. No severe complications were reported in rTMS research. The study documented minor to moderate symptoms—such as mild headaches, lightheadedness, and fatigue—that were transient, well-tolerated, and resolved promptly after discontinuing treatment. These findings suggest that rTMS may be a safer alternative to pharmacotherapy for patients with ID.

Overall, our meta-analysis indicates that rTMS provides both short- and long-term benefits for patients with ID and has examined factors that influence treatment outcomes. Our findings collectively support shifting rTMS therapy from a generally effective approach to a more targeted, stratified approach. This strategy primarily recommends using LF-rTMS targeting the right DLPFC or, for most patients with ID, cTBS. Through indirect and mixed-treatment comparisons of various interventions in the study, the treatment protocol can be personalized based on individual factors such as available time and treatment response, and may be effectively combined with pharmacotherapy, cognitive-behavioral therapy, and other therapeutic techniques. Furthermore, this meta-analysis provides new insights into how rTMS affects objective sleep metrics, including sleep efficiency (SE), sleep onset latency (SOL), total sleep time (TST), wake after sleep onset (WASO), and number of awakenings (NA). For complex cases refractory to standard therapy, exploring sequential multi-target stimulation—such as targeting the bilateral frontal and parietal areas and the temporoparietal-occipital (TPO) region—along with integrated interventions (acupuncture, music therapy, tuina massage, and meditation) offers a promising personalized approach for future research and clinical practice. Additionally, our study focused exclusively on primary insomnia. A comprehensive review of the effectiveness and safety profile of rTMS was conducted through sensitivity and subgroup analyses, assessing its performance both as a standalone treatment and when combined with other therapies.

Current pharmacological strategies for managing sleep disorders target multiple mechanisms, mainly by modulating key neurotransmitter systems and blocking receptors that promote wakefulness. Benzodiazepines (triazolam, temazepam) and Z-drugs (zolpidem, eszopiclone) enhance the effect of gamma-aminobutyric acid (GABA), the main inhibitory neurotransmitter in the brain, thereby broadly suppressing neuronal activity and promoting sleep (Palagini and Bianchini, 2022). In contrast, orexin receptor antagonists prevent binding of wake-promoting orexin (hypocretin) neuropeptides to their receptors, reducing hyperarousal and supporting a more natural sleep pattern (Yue et al., 2023). Melatonin receptor agonists (ramelteon) activate melatonin receptors within the suprachiasmatic nucleus to modulate circadian rhythms and facilitate sleep initiation, especially beneficial for circadian rhythm-related sleep disorders (Ren et al., 2025). Additionally, sedating antihistamines (diphenhydramine) and certain sedative antidepressants (trazodone) mainly work through antagonism of histamine H₁ receptors, leading to drowsiness (Roehrs and Roth, 2012). However, these pharmacological approaches have significant limitations. The complex, multifaceted causes of sleep disorders indicate that single-target agents often cannot address all underlying factors. While medications mainly ease the symptom of “inability to sleep,” they often fail to treat the hidden psychological, behavioral, or environmental reasons behind insomnia. Prolonged use of benzodiazepines and Z-hypnotics can cause physical dependence and drug tolerance, leading to the need for higher doses to maintain effectiveness (Pétursson, 1994). Many sedative-hypnotics, especially GABAergic drugs, impair memory, attention, and motor skills—effects that are especially noticeable in older adults (Zisapel, 2012). In summary, pharmacotherapy for insomnia is like a “forced shutdown”; it can put the system into a resting state, but does not fix the root causes of sleep problems. Its limitations lie in its broad actions, limited engagement with targets, and neglect of key underlying processes.

Unlike pharmacological interventions, rTMS does not act through a single “hypnotic drug” mechanism; rather, it improves sleep by modulating brain function at multiple levels. The primary ways rTMS helps to enhance sleep disorders include: (1) Neuroplasticity modulation happens by stimulating cortices involved in sleep regulation. Evidence indicates that rTMS can alter the strength of connections between brain regions. It decreases hyperactivity in wake-related areas, modulates cortical excitability, reverses abnormal neural activity patterns, reduces hyperarousal associated with insomnia, and facilitates sleep initiation (Lanza et al., 2023). (2) Sleep is regulated by the coordinated activity of widespread brain systems, including the DMN, executive control network (ECN), and visual network (VN). Patients with insomnia often exhibit disrupted connectivity within these networks (Ma et al., 2018; Wei et al., 2020). For instance, during the rest phase, patients with insomnia show increased neural connectivity between the premotor and visual cortices. This suggests that individuals with insomnia may allocate additional cortical resources in visuomotor areas to redirect attention, compared with individuals with normal sleep patterns (Perrier et al., 2023). Abnormal communication within the DMN and primary sensory systems (including visual, auditory, and motor) may lead to increased brain activity in individuals with insomnia (Killgore et al., 2013). The study by Li et al. (2022) showed that 1 Hz rTMS over the left DLPFC may help normalize hyperexcited functional connections between the left DLPFC and the right SFG (Superior Frontal Gyrus) in patients with insomnia. Xin et al. (2025) reported that rTMS promotes the normalization of disrupted neural networks by modulating dynamic connectivity among large-scale brain systems and inducing remote neural effects. (3) Studies on neurotransmitter regulation show that rTMS increases GABA, the brain’s main inhibitory neurotransmitter; boosts BDNF expression to support neuronal health and plasticity; and adjusts monoaminergic signaling—including serotonin, norepinephrine, and dopamine—collectively affecting mood, arousal, and sleep patterns (Feng et al., 2019). Unlike pharmacological agents, which typically induce strong receptor activation or blockade, rTMS promotes a more balanced, natural modulation of neurotransmitter activity. (4) Preliminary findings suggest that rTMS, especially when targeting pineal-related pathways, may affect melatonin secretion, helping to regulate disrupted circadian rhythms (Jiang et al., 2013). Overall, rTMS is a well-tolerated treatment for patients with insomnia disorder, with few severe complications reported in studies. The observed improvements in sleep highlight its safety and clinical effectiveness (Lanza et al., 2023; Zhu et al., 2024). These results confirm the effectiveness of rTMS in treating sleep disturbances and provide greater insight into its potential mechanisms and determinants.

### In the subgroup analysis

#### Disorder duration

Evidence indicates that the duration of illness in ID patients significantly affects rTMS therapeutic outcomes by influencing brain plasticity and the severity of the pathology. In cases with a short disease duration (<1–2 years), neural networks have not yet stabilized in pathological patterns. The brain retains greater neuroplastic potential, and pathological changes are relatively limited, primarily manifesting as dysregulation of neural circuitry (Centorino et al., 2020). However, our subgroup analysis showed that rTMS significantly improved the total PSQI score in both patient groups (>6 vs. ≤6 months), with a slightly greater effect in patients with ID <6 months (WMD: −3.22, 95% CI: −4.92, −1.51; *p* = 0.34). Several factors may explain this difference. First, the cutoff points used to define disease duration in the subgroup analysis may have been imprecise. Second, the small number of available studies resulted in comparisons with limited statistical power due to small sample sizes. Third, outcome assessment in this subgroup relied solely on the total PSQI score, without validation through other objective measures.

#### Treatment duration

In our meta-analysis, treatment duration for the real rTMS and sham groups was primarily 2 or 4 weeks. Subgroup analysis by treatment duration showed that both durations (≤2 vs. > 2 weeks) produced significant improvements in sleep. However, high heterogeneity was observed among studies with treatment durations >2 weeks. This variability may be due to the new stimulation site (Cz) and the older average age (73.15 ± 7.22 years) in these studies; after excluding 陆艺 et al. (2023) and 张雷鸣 et al. (2023), heterogeneity was significantly reduced. Despite a lack of statistical significance between courses ≤2 weeks and those >2 weeks, this further suggests that treatment duration may not be the primary factor influencing the efficacy of rTMS for insomnia disorder. For example, a study by Liang Gong et al. reported a remission rate of 68.18% for insomnia when MRI-guided neuronavigation was used to precisely target the right DLPFC, highlighting the importance of accurate targeting in modulating DLPFC network reorganization (Gong et al., 2025). As treatment duration increases, therapeutic effects may gradually reach a plateau. In other words, after a certain point, extending treatment may not yield additional meaningful improvements. For example, one study found that although rTMS showed significant benefits within the first 2–3 weeks, the rate of improvement slowed down thereafter (Wu et al., 2021). Currently, evidence regarding the optimal stratification of treatment duration remains limited, and more rigorous RCTs are needed to clarify its effectiveness.

#### rTMS sites

Stimulation-site-based subgroup analysis revealed that, among the studies included in our meta-analysis, 11 used the R-DLPFC as the main stimulation target, while the other studies examined different targets, such as the L-DLPFC, DMPFC, bilateral frontal and parietal areas, Cz, and TPO. Given the sparse research literature, we compared efficacy only between R-DLPFC and L-DLPFC/DMPFC stimulation. Both sites significantly improved sleep-related outcomes, as measured by PSQI and ISI total scores; however, the groups showed no statistically significant difference (*p* > 0.05). The R-DLPFC group demonstrated marginally higher ISI score gains than the L-DLPFC/DMPFC cohort. The following points may assist in interpreting these findings: The DLPFC is commonly targeted with rTMS for treating various neuropsychiatric conditions, such as depression (Eshel et al., 2020). The DLPFC, a pivotal node within the frontoparietal network, significantly contributes to the integration of cognitive and emotional functions (Gong et al., 2020). Neuroimaging reveals heightened DLPFC activity in insomniacs compared to normal sleepers (Spiegelhalder et al., 2013). Consequently, LF-rTMS targeting the DLPFC emerges as a viable intervention for insomnia. In clinical settings, the typical approach is to administer low-frequency rTMS to the R-DLPFC, thereby inducing cortical hyperpolarization. This, in turn, reduces local neural activity and helps restore proper communication between the prefrontal area and more distant brain regions that have become desynchronized (Hildebrand et al., 2024). According to Pang and colleagues’ findings, lower regional homogeneity (ReHo) in regions including the L-DLPFC and DMPFC is inversely related to individuals’ subjective sleep quality ratings (Pang et al., 2018). The L-DLPFC/DMPFC, a key component of the DMN, plays a significant role in the complexities of insomnia. It could be an important target for therapeutic intervention. For example, 刘 et al. (2024) demonstrated that activating just one node in the DMN—the prefrontal cortex—can effectively reduce activity in that specific area. This local suppression can then spread through the network, reducing broader cortical excitability—a process that might contribute to the decrease in metabolic activity observed in this region during sleep onset. As a result, stimulation of a single node within the broader DMN might trigger substantial shifts in functional connectivity throughout the entire system (Hildebrand et al., 2024). Future clinical research should carefully evaluate the efficacy of rTMS across different sites in patients with ID.

#### Differences in basic treatment

In the comparative analysis of subgroups by concomitant therapies, four studies investigated rTMS combined with medication versus medication alone. Subgroup analysis demonstrated that the combined treatment significantly enhanced sleep quality, as measured by the PSQI total score, compared with medication alone (WMD: −2.77; 95% CI: −4.62, −0.92; *p* = 0.03). Since the mechanisms of rTMS and pharmacotherapy in treating insomnia have been previously discussed, this finding aligns with our expectations. The next section will explore the potential benefits and limitations of combining medication with rTMS for insomnia disorder. Potential Benefits: First, the combination of rTMS and pharmacotherapy may produce synergistic effects, thereby enhancing overall treatment efficacy. Pharmacological agents (sedative-hypnotics) can rapidly modulate neurotransmitter levels, providing short-term symptom relief. In contrast, rTMS modulates cortical excitability and neuroplasticity within brain networks. This mechanistic complementarity may lead to more sustained and fundamental improvements in sleep regulation (Wu et al., 2021; De Crescenzo et al., 2022). For example, Lin et al. (2023) demonstrated that combined rTMS and medication was significantly better than medication alone in improving PSQI, increasing TST, and increasing the proportion of slow-wave sleep, indicating more comprehensive symptom improvement. Second, for patients with treatment-resistant or recurrent insomnia who respond inadequately to conventional drug therapy, the combined approach offers a new therapeutic option. Integrating interventions with distinct mechanisms may help overcome the limitations of monotherapy. Third, theoretically, as the cumulative effects of rTMS help stabilize brain function, combination therapy could create an opportunity to gradually reduce medication dosage. This could mitigate the dangers linked to extended medication treatments, such as dependence, tolerance, and daytime drowsiness, facilitating a transition from “pharmacological maintenance” to “maintenance via neuromodulation. For example, Zheng et al. (2025) investigated the feasibility of combining rTMS with dexmedetomidine for chronic insomnia. Their study demonstrated that the administration of LF-rTMS significantly reduced the required DEX dose by the end of the treatment course, whereas the combined regimen was well tolerated and associated with a favorable prognosis (Zheng et al., 2025).

### Key limitations

First, although the side-effect profiles of the two modalities differ, their combination may increase the risk of adverse effects, including dizziness, headache, and fatigue, particularly during the initial treatment phase (Zheng et al., 2025). Furthermore, therapeutic responses vary among individuals, and not all patients experience synergistic (“1 + 1 > 2”) benefits. Additionally, there is currently no universally accepted “gold-standard” protocol for combining these treatments—such as the order of modalities (which to start with), the timing of medication tapering, and how to adjust rTMS parameters in conjunction with pharmacotherapy. Clinical decision-making largely relies on physician experience and individual patient responses, resulting in a complex, nonstandardized treatment approach that may lead to overtreatment in mild cases. Thirdly, for patients with mild-to-moderate, first-onset insomnia, rTMS or medication alone may be sufficiently effective. Combination therapy seems more appropriate for moderate-to-severe, treatment-resistant cases, or those with complex comorbidities. In summary, combining pharmacotherapy and rTMS acts as a “double-edged sword.” While it offers new hope for patients with complex, refractory insomnia, it also introduces increased therapeutic complexity and requires careful clinical judgment. This approach selection requires a comprehensive evaluation of the patient’s individual health status—including insomnia duration, comorbidities, and prior treatment history—followed by fully informed, shared decision-making and tailored to the individual. Finally, we compared the therapeutic effects of rTMS with other treatments (including CBT-I, psychotherapy, AMFT, and tDCS). After excluding the study by 陶晟 et al. (2020) due to its high heterogeneity, the analysis showed that rTMS produced a significantly greater improvement in sleep outcomes than the other treatments (WMD: −1.33; 95% CI: −2.56, −1.10; *p* = 0.03). Our results highlight that although rTMS appears more effective, other treatments still confer measurable sleep benefits. This supports their clinical relevance and warrants further investigation. CBT-I is a structured, non-drug intervention that employs educational, cognitive, and behavioral strategies—such as sleep restriction and cognitive restructuring—to correct unhealthy sleep-related mindsets and perceptions, lessen excessive worry and rumination concerning sleep, and improve the homeostatic “sleep drive,” which is viewed as the most essential factor for effective treatment (Altena et al., 2023). For example, according to Barroso et al.’ (2024) comprehensive analysis, CBT-I is highly effective in reducing Insomnia Severity Index scores and serves as a safeguard against acute insomnia progressing to a chronic condition. Unfortunately, the shortage of qualified mental health professionals and the labor-intensive nature of CBT-I have hindered both scientific research and widespread adoption in everyday practice. CBT-I generally includes four recommended therapeutic components. However, interactions among certain elements may not always be synergistic; they may be antagonistic. The overall quality of evidence supporting specific combinations of components is typically rated as moderate to low (Furukawa et al., 2024). Therefore, larger and more carefully designed trials are still necessary to determine whether certain combinations are better than others or just the individual components alone. The field of transcranial electrical stimulation, primarily including tDCS and tACS (Transcranial alternating current stimulation), has seen increasing activity in clinical trials. While tDCS modulates neuronal excitability, rTMS elicits action potentials and thus engages distinct neural pathways (Zhou et al., 2024). Clinical trials have demonstrated that in patients with insomnia, tDCS can improve sleep by modulating GABA and glutamate concentrations, modifying membrane polarization and cortical responsiveness, and enhancing cerebral oxyhemoglobin levels, along with other hemodynamic metrics (Stagg and Nitsche, 2011; Frase et al., 2016). Nonetheless, the use of tDCS to improve sleep quality remains debated. Lukas Frase and his colleagues’ 2019 study found no significant differences in sleep consistency or sleep architecture between patients and healthy subjects. In contrast, rTMS works through more complex, multi-level mechanisms (Frase et al., 2019). Therefore, there is a need to develop adaptive tDCS protocols to examine combined therapeutic impacts with rTMS, thereby better meeting the needs of different patient subtypes. Additionally, this meta-analysis included studies on Alternating Magnetic Field Therapy (AMFT), which applies external alternating magnetic fields to the human body and is postulated to modulate neural electrical activity or local blood circulation, thereby regulating the sleep–wake rhythm (陶 et al., 2020). However, solid clinical evidence supporting AMFT for treating insomnia remains limited. In the current evidence-based medical framework, AMFT has not been included in major national or international guidelines for treating insomnia. Therefore, further well-designed clinical trials are necessary to confirm its standalone effectiveness and clarify its underlying mechanisms.

In our meta-analysis, some outcomes showed notable heterogeneity. We performed planned subgroup and sensitivity analyses to address this heterogeneity. Our results indicate that factors such as patient age, rTMS stimulation site, and different types of concomitant pharmacotherapy may explain the variability. Differences in effect estimates across studies probably also contributed. Additionally, the natural variability in sleep and rTMS delivery should be recognized as a possible confounder.

### Limitations and future research

This study has several limitations: first, the small number of studies on rTMS for ID; the meta-analysis included only 19 studies (23 trials), resulting in a limited sample size. Second, limited research prevented a comprehensive analysis of the rTMS stimulation site and age-related effects. Third, only four studies reported follow-up data, leaving the long-term effectiveness of rTMS for insomnia unclear and underscoring the need for further research. Fourth, evidence on objective clinical outcomes related to sleep assessment remains limited, as only two studies reported multimodal measures—including functional connectivity, resting-state EEG, and serum inflammatory markers—while polysomnography (PSG) was not widely used. Nonetheless, these preliminary findings identify promising biomarker candidates. Finally, as the studies under review were available only in Chinese or English, pertinent research in other languages might have been inadvertently omitted. Based on these findings, several directions for future research are suggested. Since rTMS for sleep disorders is a significant treatment option, large-scale, multicenter, RCTs are necessary to strengthen the current evidence. Further research is needed to develop standardized rTMS protocols and to integrate them with other therapies. Additionally, more clarification is needed on how the objective evaluation parameters relate to therapeutic outcomes in sleep disorders. Finally, the long-term effectiveness of rTMS should be confirmed through long-term studies.

## Conclusion

In summary, this meta-analysis provides robust evidence of a notable improvement in sleep metrics and patterns among individuals with sleep disturbances following rTMS treatment. Compared to rTMS monotherapy, combining rTMS with medication may provide extra sleep benefits. Given the potential for additional side effects and the absence of standardized protocols, the clinical use of combination therapy should be approached cautiously and tailored to each patient’s profile. Further RCTs are needed to validate and standardize such combined regimens. This meta-analysis includes the largest and most recent cohort to date (*n* = 1,690) investigating rTMS for insomnia disorder. In addition to comparing real rTMS with sham stimulation, four *a priori*-defined subgroup analyses were performed to identify possible effect modulators—including disease duration, rTMS treatment course, stimulation site, and concomitant medication use—and their possible clinical mechanisms. Importantly, no serious adverse events were associated with rTMS, supporting its safe profile as a treatment for sleep disorders. Common protocols typically involve LF-rTMS targeting the R-DLPFC. Collectively, these findings provide valuable guidance for the development of future therapeutic strategies.

## Data Availability

The original contributions presented in the study are included in the article/Supplementary material, further inquiries can be directed to the corresponding authors.
