# Supplementary material for: Efficacy of repetitive transcranial magnetic stimulation for insomnia disorder: a systematic review and meta-analysis of randomized controlled trials
Source: Front Neurosci. 2026 Jun 1;20:1816963. doi: 10.3389/fnins.2026.1816963 (PMC13265460; doi:10.3389/fnins.2026.1816963)
Supplement: Supplementary file 1 [file Supplementary_file_1.DOCX]

**Supplementary material 1: Search strategy**

| Database | Date | Terms | Results |
| --- | --- | --- | --- |
| MEDLINE  PubMed | 2025-10-13 | #1: Transcranial Magnetic Stimulation |  |
|  |  | "Transcranial Magnetic Stimulation"[MeSH Terms] OR ("Transcranial Magnetic Stimulation"[MeSH Terms] OR ("transcranial"[All Fields] AND "magnetic"[All Fields] AND "stimulation"[All Fields]) OR "Transcranial Magnetic Stimulation"[All Fields] OR ("magnetic"[All Fields] AND "stimulations"[All Fields] AND "transcranial"[All Fields]) OR ("Transcranial Magnetic Stimulation"[MeSH Terms] OR ("transcranial"[All Fields] AND "magnetic"[All Fields] AND "stimulation"[All Fields]) OR "Transcranial Magnetic Stimulation"[All Fields] OR ("magnetic"[All Fields] AND "stimulation"[All Fields] AND "transcranial"[All Fields]) OR "magnetic stimulation transcranial"[All Fields]) OR ("Transcranial Magnetic Stimulation"[MeSH Terms] OR ("transcranial"[All Fields] AND "magnetic"[All Fields] AND "stimulation"[All Fields]) OR "Transcranial Magnetic Stimulation"[All Fields] OR ("stimulations"[All Fields] AND "transcranial"[All Fields] AND "magnetic"[All Fields])) OR ("Transcranial Magnetic Stimulation"[MeSH Terms] OR ("transcranial"[All Fields] AND "magnetic"[All Fields] AND "stimulation"[All Fields]) OR "Transcranial Magnetic Stimulation"[All Fields] OR ("stimulation"[All Fields] AND "transcranial"[All Fields] AND "magnetic"[All Fields]) OR "stimulation transcranial magnetic"[All Fields]) OR ("Transcranial Magnetic Stimulation"[MeSH Terms] OR ("transcranial"[All Fields] AND "magnetic"[All Fields] AND "stimulation"[All Fields]) OR "Transcranial Magnetic Stimulation"[All Fields] OR ("transcranial"[All Fields] AND "magnetic"[All Fields] AND "stimulations"[All Fields]) OR "transcranial magnetic stimulations"[All Fields]) OR ("Transcranial Magnetic Stimulation"[MeSH Terms] OR ("transcranial"[All Fields] AND "magnetic"[All Fields] AND "stimulation"[All Fields]) OR "Transcranial Magnetic Stimulation"[All Fields] OR ("transcranial"[All Fields] AND "magnetic"[All Fields] AND "stimulation"[All Fields] AND "paired"[All Fields] AND "pulse"[All Fields]) OR "transcranial magnetic stimulation paired pulse"[All Fields]) OR ("Transcranial Magnetic Stimulation"[MeSH Terms] OR ("transcranial"[All Fields] AND "magnetic"[All Fields] AND "stimulation"[All Fields]) OR "Transcranial Magnetic Stimulation"[All Fields] OR ("transcranial"[All Fields] AND "magnetic"[All Fields] AND "stimulation"[All Fields] AND "repetitive"[All Fields]) OR "transcranial magnetic stimulation repetitive"[All Fields]) OR ("Transcranial Magnetic Stimulation"[MeSH Terms] OR ("transcranial"[All Fields] AND "magnetic"[All Fields] AND "stimulation"[All Fields]) OR "Transcranial Magnetic Stimulation"[All Fields] OR ("transcranial"[All Fields] AND "magnetic"[All Fields] AND "stimulation"[All Fields] AND "single"[All Fields] AND "pulse"[All Fields]) OR "transcranial magnetic stimulation single pulse"[All Fields])) | 26978 |
|  |  | #2：Sleep Initiation and Maintenance Disorders |  |
|  |  | ("Sleep Initiation and Maintenance Disorders"[Mesh]) OR (DIMS (Disorders of Initiating and Maintaining Sleep) OR Disorders of Initiating and Maintaining Sleep OR Sleeplessness OR Insomnia Disorder OR Insomnia Disorders OR Insomnia OR Insomnias OR Chronic Insomnia OR Insomnia, Chronic OR Early Awakening OR Awakening, Early OR Nonorganic Insomnia OR Insomnia, Nonorganic OR Primary Insomnia OR Insomnia, Primary OR Psychophysiological Insomnia OR Insomnia, Psychophysiological OR Rebound Insomnia OR Insomnia, Rebound OR Secondary Insomnia OR Insomnia, Secondary OR Sleep Initiation Dysfunction OR Dysfunction, Sleep Initiation OR Dysfunctions, Sleep Initiation OR Sleep Initiation Dysfunctions OR Transient Insomnia OR Insomnia, Transient) | 44939 |
|  |  | #1 AND#2 | 155 |
|  |  | Filters applied: Randomized Controlled Trial. | **39** |
| The Cochrane library | 2025-10-13 | Transcranial Magnetic Stimulation |  |
|  |  | #1:MeSH descriptor: [Transcranial Magnetic Stimulation] explode all trees  #2:(Transcranial Magnetic Stimulation):ti,ab,kw OR (Magnetic Stimulations, Transcranial):ti,ab,kw OR (Magnetic Stimulation, Transcranial):ti,ab,kw OR (Stimulations, Transcranial Magnetic):ti,ab,kw OR (Stimulation, Transcranial Magnetic):ti,ab,kw  #3:(Transcranial Magnetic Stimulations):ti,ab,kw OR (Transcranial Magnetic Stimulation, Paired Pulse):ti,ab,kw OR (Transcranial Magnetic Stimulation, Repetitive):ti,ab,kw OR (Transcranial Magnetic Stimulation, Single Pulse):ti,ab,kw  #4:#1 OR #2 OR #3 | 9589 |
|  |  | **Sleep Initiation and Maintenance Disorders** |  |
|  |  | #5: MeSH descriptor: [Sleep Initiation and Maintenance Disorders] explode all trees  #6: (Insomnia, Secondary or Secondary Insomnia or Psychophysiological Insomnia or Insomnia, Psychophysiological or DIMS (Disorders of Initiating and Maintaining Sleep) or Disorders of Initiating and Maintaining Sleep or Insomnia, Nonorganic or Nonorganic Insomnia or Primary Insomnia or Insomnia, Primary or Insomnia, Chronic or Chronic Insomnia or Transient Insomnia or Insomnia, Transient or Insomnias or Sleeplessness or Insomnia or Insomnia Disorders or Insomnia Disorder or Early Awakening or Awakening, Early or Sleep Initiation Dysfunction or Dysfunctions, Sleep Initiation or Dysfunction, Sleep Initiation or Sleep Initiation Dysfunctions or Rebound Insomnia or Insomnia, Rebound):ti,ab,kw  #7: #5 OR #6 | 18223 |
|  |  | #8: #4 AND #7 | 184 |
|  |  | Filters applied: **Randomized Controlled Trial.** | **184** |
| **Embase** | **2025-10-13** | Transcranial Magnetic Stimulation |  |
|  |  | #1:'transcranial magnetic stimulation'/exp' OR 'repetitive transcranial magnetic stimulation'/exp OR 'magnetic stimulations, transcranial':ti,ab,kw OR 'magnetic stimulation, transcranial':ti,ab,kw OR 'stimulations, transcranial magnetic':ti,ab,kw OR 'stimulation, transcranial magnetic':ti,ab,kw OR 'transcranial magnetic stimulations':ti,ab,kw OR 'transcranial magnetic stimulation, paired pulse':ti,ab,kw OR 'transcranial magnetic stimulation, repetitive':ti,ab,kw OR 'transcranial magnetic stimulation, single pulse':ti,ab,kw | 41326 |
|  |  | insomnia |  |
|  |  | #2: 'insomnia'/exp OR 'agrypnia':ti,ab,kw OR 'disorder of sleep initiation and maintenance':ti,ab,kw OR 'disorders of sleep initiation and maintenance':ti,ab,kw OR 'hyposomnia':ti,ab,kw OR 'hyposomnias':ti,ab,kw OR 'insomnia disorder':ti,ab,kw OR 'insomnia disorders':ti,ab,kw OR 'insomnias':ti,ab,kw OR 'sleep initiation and maintenance disorder':ti,ab,kw OR 'sleep initiation and maintenance disorders':ti,ab,kw OR 'sleep initiation/maintenance disorder':ti,ab,kw OR 'sleeplessness':ti,ab,kw OR 'insomnia':ti,ab,kw | 116636 |
|  |  | #1 and #2 | 633 |
|  |  | **Filters applied: Randomized Controlled Trial.** | **405** |
| **Web of Science** | **2025-10-13** | **Transcranial Magnetic Stimulation** |  |
|  |  | #1: (TS=(repetitive transcranial magnetic stimulation) OR TS=(transcranial magnetic stimulation) OR TS=(magnetic stimulations, transcranial) OR TS=(magnetic stimulation, transcranial) OR TS=(stimulations, transcranial magnetic) OR TS=(stimulation, transcranial magnetic) OR TS=(transcranial magnetic stimulations) OR TS=(transcranial magnetic stimulation, paired pulse) OR TS=(transcranial magnetic stimulation, repetitive) OR TS=(transcranial magnetic stimulation, single pulse)) | 45754 |
|  |  | **Sleep Initiation and Maintenance Disorders** |  |
|  |  | #2: (TS=(**Sleep Initiation and Maintenance Disorders) OR** TS=(DIMS (Disorders of Initiating and Maintaining Sleep)) OR TS=(Disorders of Initiating and Maintaining Sleep) OR TS=(Insomnia Disorder) OR TS=(Sleeplessness) OR TS=(Insomnia Disorders) OR TS=(Insomnia) OR TS=(Insomnias) OR TS=(Chronic Insomnia) OR TS=(Insomnia, Chronic) OR TS=(Early Awakening) OR TS=(Awakening, Early) OR TS=(Nonorganic Insomnia) OR TS=(Insomnia, Nonorganic) OR TS=(Primary Insomnia) OR TS=(Insomnia, Primary) OR TS=(Psychophysiological Insomnia) OR TS=(Insomnia, Psychophysiological) OR TS=(Rebound Insomnia) OR TS=(Insomnia, Rebound) OR TS=(Secondary Insomnia) OR TS=(Insomnia, Secondary) OR TS=(Sleep Initiation Dysfunction) OR TS=( Dysfunction, Sleep Initiation) OR TS=(Dysfunctions, Sleep Initiation) OR TS=(Sleep Initiation Dysfunctions) OR TS=(Transient Insomnia) OR TS=(Insomnia, Transient)) | 83309 |
|  |  | **#1 AND #2 Filters applied: Filters applied: Randomized Controlled Trial AND Clinical Trial** | **91** |
| ***China National Knowledge Infrastructure*** | **2025-10-13** | **重复经颅磁刺激** |  |
|  |  | **#1：**SU%=('重复经颅磁刺激'+'经颅磁刺激'+'无创性脑刺激'+'非侵入性脑刺激'+'间歇性θ节律刺激'+'间歇性θ短阵快速脉冲'+'Theta爆发式磁刺激'+'单脉冲经颅磁刺激'+'成对脉冲经颅磁刺激'+'无创神经调控技术'+'高频重复经颅磁刺激'+'低频重复经颅磁刺激'+'θ节律刺激'+'脑调控') | 8195 |
|  |  | **入睡和睡眠障碍** |  |
|  |  | **#2：**SU%=('入睡和睡眠障碍'+'反跳性失眠症'+'继发性失眠'+'睡眠起始功能障碍'+'失眠'+'失眠症'+'慢性失眠'+'早醒'+'非器质性失眠症'+'原发性失眠'+'入睡和睡眠失调'+'DIMS'+'心理生理性失眠'+'暂时性失眠'+'睡眠困难'+'不寐'+'入睡难'+'不得卧'+'目不冥') | 46000 |
|  |  | **#3：#1 AND #2** | 300 |
|  |  | **Filters applied: Randomized Controlled Trial.** | **300** |
| ***Wanfang Database*** | **2025-10-13** | **重复经颅磁刺激** |  |
|  |  | **#1：**（主题:(经颅磁刺激 or 重复经颅磁刺激 or 经颅磁刺激 or 无创性脑刺激 or 非侵入性脑刺激 or 间歇性θ节律刺激 or 间歇性θ短阵快速脉冲 or 单脉冲经颅磁刺激 or 无创神经调控技术 or 高频重复经颅磁刺激 or 低频重复经颅磁刺激 or 脑调控) | 80968 |
|  |  | **入睡和睡眠障碍** |  |
|  |  | **#2：**主题:(入睡和睡眠障碍 or 反跳性失眠症 or 继发性失眠 or 睡眠起始功能障碍 or 失眠 or 失眠症 or 慢性失眠 or 早醒 or 非器质性失眠症 or 原发性失眠 or 入睡和睡眠失调 or DIMS or 心理生理性失眠 or 暂时性失眠 or 睡眠困难 or 不寐 or 入睡难 or 不得卧 or 目不冥) | 83793 |
|  |  | **#3：#1 AND #2** | 638 |
|  |  | **Filters applied: Randomized Controlled Trial.** | **490** |
